# Integrative analysis of metabolome and gut microbiota in diet-induced hyperlipidemic rats treated with berberine compounds

**DOI:** 10.1186/s12967-016-0987-5

**Published:** 2016-08-05

**Authors:** Meng Li, Xiangbing Shu, Hanchen Xu, Chunlei Zhang, Lili Yang, Li Zhang, Guang Ji

**Affiliations:** 1 Institute of Digestive Diseases, China-Canada Center of Research for Digestive Diseases (ccCRDD), Longhua Hospital, Shanghai University of Traditional Chinese Medicine, Shanghai, 200032 China; 2 E-institute of Shanghai Municipal Education Commission, Shanghai University of Traditional Chinese Medicine, Shanghai, 201203 China

**Keywords:** Berberine compounds, Integrative metabolomics, Gut microbiota, Hyperlipidemia

## Abstract

**Background:**

Hyperlipidemia is a major component of metabolic syndrome, and often predicts cardiovascular diseases. We developed a new therapeutic agent berberine compounds (BC), consisting of berberine, oryzanol and vitamin B_6_, and determined their anti-hyperlipidemia activity and underlying mechanisms.

**Methods:**

Male Wistar rats were fed a high fat diet (HFD) to induce hyperlipidemia, and then given BC orally for 4 weeks. Body weight and food intake were recorded weekly, and lipid profiles in serum were determined biochemically. Metabolites in serum, urine, liver and feces were analyzed by GC–MS, and the structure of microbiota was determined by 16S rDNA sequencing.

**Results:**

Lipid lowering was observed in the hyperlipidemic rats upon BC treatment without apparent adverse side effects. Metabolomics analysis indicated that the BC treatment resulted in increased pyruvic acid, serotonin, and ketogenic and glycogenic amino acid levels in the serum, increased pyridoxine and 4-pyridoxic acid in the urine, decreased hypotaurine and methionine in the liver, and increased putrescine and decreased deoxycholate and lithocholate in feces. The BC treatment also resulted in an enrichment of beneficial bacteria (e.g. *Bacteroides*, *Blautia*) and a decrease in *Escherichia*.

**Conclusions:**

The lipid lowering effect of BC treatment in hyperlipidemic rats is associated with a global change in the metabolism of lipids, carbohydrates and amino acids, as well as the structure of microbiota.

**Electronic supplementary material:**

The online version of this article (doi:10.1186/s12967-016-0987-5) contains supplementary material, which is available to authorized users.

## Background

Hyperlipidemia is a consternation of several plasma lipoprotein abnormalities, including increased triglyceride (TG) and/or total cholesterol (TC), low-density lipoprotein cholesterol (LDL-c), and decreased high-density lipoprotein cholesterol (HDL-c) in serum [1]. Regarded as a modifiable risk factor for metabolic diseases including nonalcoholic fatty liver disease, type 2 diabetes mellitus and cardiovascular disease [2, 3], hyperlipidemia is becoming an important public health concern with increased incidence and prevalence [1]. Commonly used medications for hyperlipidemia include statins, fibrates, bile acid sequestrants, nicotinic acid, and cholesterol absorption inhibitors [4]. Unfortunately, many medications have significant adverse effects, such as statin myopathy, liver injury, and sleep disturbance [5]. Hence, new drugs of effectiveness and safety remain to be explored.

Some natural products that been used in traditional Chinese medicine show remarkable anti-hyperlipidemia efficacies. For instance, the alkaloid berberine (BBR), a quaternary ammonium salt isolated from *Rhizoma coptidis* (Huanglian) [6] used as a broad-spectrum anti-microbial medicine, possesses an anti-hyperlipidemic potential [7, 8]. Both pre-clinical and clinical studies have demonstrated beneficial effects of BBR in the management of hyperlipidemia [9, 10]. However, the low intestinal bioavailability of BBR [11], demands a high dose and/or long-term treatment, which often elicit unwanted side effects (e.g. constipation, nausea, and abdominal distension) [12].

We have developed a new agent, designated BBR compounds (BC), consisting of BBR, oryzanol and vitamin B_6_. Oryzanol is a particular bioactive compound found in rice bran that has been shown to exert an effect on lipid metabolism [13–15]. Vitamin B_6_ is a co-enzyme that participates in various metabolic activities [16]. In addition, oryzanol and vitamin B_6_ appear to exert an effect on metabolic pathways that are shared with BBR [14–16].

In this study, we first examined the effect of BC in alleviating hyperlipidemia using a diet-induced rat model, and determined metabolome using gas chromatography/mass spectrometry (GC/MS)-based approach [17–20]. Because gut microbiota play a critical role in modulating metabolism and drug bioavailability [21–23], the effect of BC treatment on the structure of gut microbiota was also determined.

## Methods

### Chemicals and reagents

BBR, γ-oryzanol, pyridoxine (vitamin B_6_), urease, pyridine, methoxylamine hydrochloride, L-phenylalanine-^13^C_9_-^15^N, dulcitol, L-leucine-^13^C_6_, L-isoleucine-^13^C_6_-^15^N, L-valine-^13^C_5_-^15^N, L-alanine-^13^C_3_-^15^N and *N*,O-bis(trimethylsilyl)-trifluoroacetamide (BSTFA) with 1 % trimethylchlorosflane (TMCS) were purchased from Sigma-Aldrich (St. Louis, MO, USA). Pure water was obtained from Millipore Alpha-Q water system (Bedford, MA, USA). Methanol and ethanol for HPLC grade were purchased from Merck Chemicals (Darmstadt, Germany). Chloroform for analytical grade from Sinopharm Chemical Reagent Company (Shanghai, China). KAPA HiFi polymerase (KAPA Biosystems, USA), Agencourt AMPure XP beads (Beckman Coulter, USA), Qubit dsDNA HS assay kit (Invitrogen, CA), Agilent high sensitivity DNA Kit (Agilent, USA), Ion PGM template OT2 200 kit, Ion PGM sequencing 200 kit v2 and Ion 316 chip kit v2 (Life Technology, CA) and QIAamp^®^ Fast DNA stool mini kit (Qiagen, Germany) were obtain for fecal microbiota analysis.

### Animal study

Male Wistar rats (8-week old, weighting 180–220 g), purchased from Shanghai SLAC Laboratory Animal Co., Ltd (Shanghai, China), were housed in a standard 12 h light/12 h dark cycle. The animal room temperature and relative humidity were 22 ± 2 °C and 60 ± 5 %, respectively. After 1-week acclimatization, rats were divided into chow diet group (n = 10) and HFD (10 % lard, 20 % sucrose, 2 % cholesterol, 1 % bile salt and 67 % standard chow) group (n = 20). Blood samples were collected randomly from tail vein of 6 rats in each group after 4 week feeding, and TC, TG, LDL-c, HDL-c and free fatty acid (FFA) level in serum were quantified. The hyperlipidemic rats were further divided into two groups, namely, untreated group (continued to supply HFD, n = 10) and BC treated group (with HFD and BC supplementation, n = 10). BC (150 mg/kg of BBR, 24 mg/kg of oryzanol and 10 mg/kg of vitamin B_6_) were suspended with 0.5 % sodium carboxy methylcellulose (CMC-Na) solution, and then administered by oral gavage for the next 4 weeks. The chow diet and HFD groups (untreated) rats were received an equal volume 0.5 % CMC-Na solution. Food intake and body weight were recorded weekly. At the end of experiment, all rats were fasted overnight, and sacrificed with 2 % sodium pentobarbital anesthesia (0.5 mL/100 g). Blood samples were drawn from the abdominal aorta, and the serum was obtained by centrifugation at 3000 rpm for 15 min at 4 °C. To collect urine and feces samples, six rats in each experimental group were housed in metabolic cages, and the samples accumulated in the metabolic cages were immediately transferred into sterile tubes. The liver samples were immediately dissected, weighted, washed with icy normal saline, snap-frozen in liquid nitrogen, and then stored at −80 °C.

All the experimental protocols were approved by the animal committee of Shanghai University of Traditional Chinese Medicine.

### Biochemical analysis of serum

The levels of TC, TG, LDL-c, HDL-c, FFA, total bilirubin (TBIL), total bile acids (TBA) and alanine aminotransferase (ALT) and aspartate aminotransferase (AST) enzymes in serum, were determined by an automatic biochemical analyzer. SPSS 17.0 statistical software (SPSS, Chicago, IL, USA) was used for data analysis. Statistical analysis was carried out using one-way analysis of variance (ANOVA). The criterion used for statistical significance was *p* < 0.05.

### Sample preparation for GC–MS

For each serum sample (20 μL), 80 μL of ice-cold methanol containing 10 μL of internal standards (0.02 mg/mL of L-phenylalanine-^13^C_9_-^15^N, 0.05 mg/mL of dulcitol, L-leucine-^13^C_6_ and L-isoleucine-^13^C_6_-^15^N, 0.1 mg/mL of L-valine-^13^C_5_-^15^N and L-alanine-^13^C_3_-^15^N) was added. For each urine sample (15 μL), 15 μL urease solution were blended and catalyzed at 37 °C for 60 min, and then 180 μL of ice-cold ethanol containing 10 μL of internal standards (0.05 mg/mL of L-phenylalanine- ^13^C_9_-^15^N and dulcitol) was added. For each liver tissue or feces sample (40 ± 5 mg), 800 μL chloroform/methanol/water (v/v/v, 2:5:2 in ice water) mixture were added and homogenized. After centrifuged at 16,000*g* for 15 min, the supernatant (100 μL) was added to a GC vial, containing 10 μL of internal standards (0.05 mg/mL of L-phenylalanine-^13^C_9_-^15^N and dulcitol).

The respective serum, urine, liver and feces samples were dried under gentle nitrogen stream. The glass vial with dry residue was added with 30 μL of 20 mg/mL methoxylamine hydrochloride in anhydrous pyridine. The resultant mixture was vortex-mixed vigorously for 30 s and incubated (37 °C for 90 min). A 30 μL of BSTFA (with 1 % TMCS) was added into the mixture and derivatized (70 °C for 60 min).

### GC/MS analysis

Each derivatized sample (1 μL) was injected using the splitless mode with an Agilent Technologies 7890A chromatograph equipped with a HP-5MS column (30 m × 0.25 mm × 0.25 μm) and Agilent Technologies 5975C inert MSD detector. The initial oven temperature was held at 70 °C for 2 min, increased to 160 °C with 6 °C/min, and then to 240 °C with 10 °C/min, and finally increase to 300 °C with 20 °C/min, constant for 6 min, with He as carrier gas (1 mL/min) and MS detection. The temperatures of injector, transfer line, and electron impact ion source were set to 250, 290, and 230 °C, respectively.

### Data processing and multivariate data analysis by GC/MS

The extraction, alignment, deconvolution, and further processing of raw GC/MS data were converted into NetCDF format via DataBridge (Perkin-Elmer, USA). The data was normalized against total peak intensities before performing univariate and multivariate statistics. Multivariate data analysis was carried out using SIMCA-P 11.0 software (Umetrics AB, Umeå, Sweden) to perform principal component analysis (PCA) where general clusters and outliers were observed. Prior to PCA, all data were mean-centered and unit variance-scaled. Subsequently, the data were subjected to partial least squares-discriminant analysis (PLS-DA) and orthogonal partial least squares-discriminant analysis (OPLS-DA) where a model was built and utilized to identify and reveal differential metabolites accountable for the separation between identified groups. The differential metabolites were determined by cross-referencing with the Golm Metabolome Database. In addition, metabolic pathway interpretation of differential metabolites was performed using the KEGG database.

### Fecal DNA extraction and pyrosequencing

For the extraction of fecal DNA, 180–220 mg each stool sample were weighed on ice and operated based on the protocol for “Isolation of DNA from stool for human DNA analysis” in the handbook provided by QIAamp DNA stool mini kit. DNA yields are determined from the concentration of DNA in the eluate, measured by absorbance at 260 nm. Purity is determined by calculating the ratio of absorbance at 260 to 280 nm, measured by NanoDrop microvolume quantitation of nucleic acids. pure DNA has an A260/A280 ratio of 1.7–1.9.

The DNA extractive from each stool sample was used as a template for the amplification of V3 region of 16S rDNA genes. The bacterial genomic DNA was PCR amplified with the forward primers (5′-TCGTCGGCAGCGTCAGATGTGTATAAGAGACAGCCTACGGGNGGCWGCAG) and the reverse primers (5′-GTCTCGTGGGCTCGGAGATGTGTATAAGAGACAGGACTACHVGGGTATCTAATCC) for the V3 hypervariable regions of the 16S rDNA gene.

The PCR condition were 95 °C for 3 min, followed by 25 cycles of 95 °C for 30 s, 55 °C for 30 s and 72 °C for 30 s, and then 72 °C for 5 min on an Eppendorf thermocycler. The PCR products were verified on a Bioanalyzer DNA 1000 chip (Agilent), and the expected size on a Bioanalyzer trace after the Amplicon PCR step is ~550 bp. Then we use AMPure XP beads to purify the 16S V3 amplicon away from free primers and primer dimer species, and quantified the sample with fluorometric quantification method that used dsDNA binding dyes. The concentrated final library was diluted to 4 nM using resuspension buffer (RSB) or 10 mM Tris pH 8.5. 5 μL of diluted DNA from each library was mixed for pooling libraries with unique indices. After samples were loaded, the MiSeq system provided instrument secondary analysis using the MiSeq Reporter software (MSR). The MSR provided several options for analyzing MiSeq sequencing data. All operations for fecal microbe analysis were performed under sterile conditions.

### Bioinformatics and statistical analysis

Raw pyrosequencing reads generated from the sequencer were quality filtered by FASTX Toolkit 0.0.13 (http://hannonlab.cshl.edu/fastx_toolkit/index.html). The high-quality valid reads were clustered into operational taxonomic units (OTUs) using Mothur (http://www.mothur.org/). The representative sequences of OTUs were used to analyze α-diversity (rarefaction curve analysis and Shannon diversity index) on the basis of their relative abundance. A heatmap was generated according to the relative abundance of OTUs by R software (http://www.R-project.org). Phylogenetic β-diversity measures such as unweighted unifrac significance test, principal coordinate analysis (PCoA) and nonmetric multidimensional scaling (NMDS) were performed using representative sequences of OTUs for each sample by the Mothur program to analyze community and phylogenesis. Taxonomy-based analyses were performed to classify taxonomically using the ribosomal database project (RDP) classifier with a 60 % bootstrap score.

## Results

### The effect of BC treatment on diet-induced hyperlipidemia in rats

**Fig. 1:**
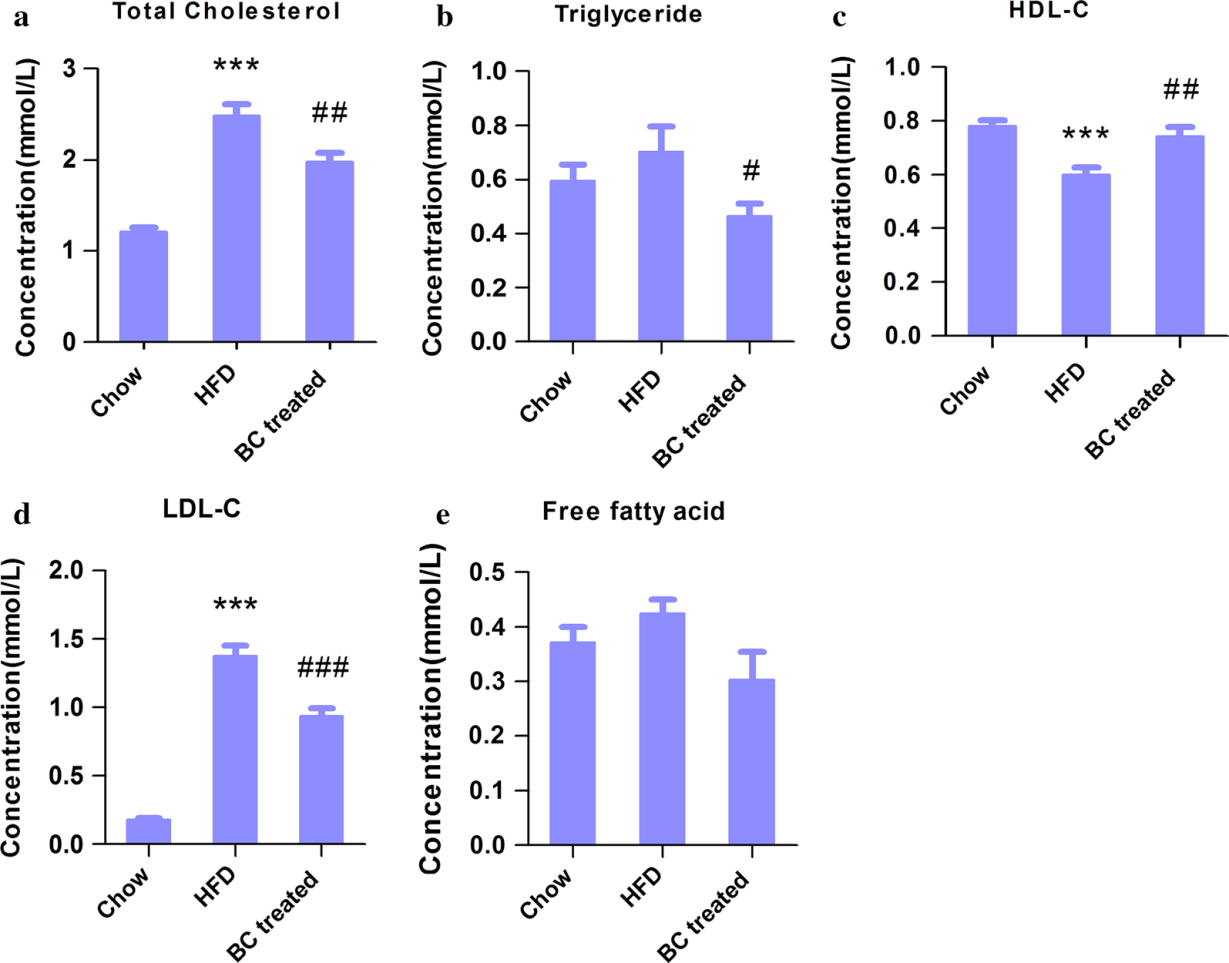
Changes of serum parameters in rats. Male Wistar rats (8 weeks of age) placed on HFD or chow diet for 8 weeks, HFD rats were divided into BC treated and untreated groups, with the 4-week intervention. At the end of experiment, blood was collected, and serum was separated, the lipid profiles were analyzed. **a** TC, **b** LDL-c, **c** HDL-c, **d** TG, **e** FFA. The data were presented as the mean ± SD. ***P < 0.001, compared with chow group; ^#^ *P* < 0.05, ^##^ *P* < 0.01 and ^###^ *P* < 0.001, compared with untreated (HFD) group

Male Wistar rats developed hyperlipidemia after 5 weeks of HFD feeding. Thus, the serum TC and LDL-c concentrations were increased whereas HDL-c concentration decreased as compared to chow diet controls (Additional file 1: Figure S1). A trend of increase in serum TG and FFA was also observed in HFD-fed group, although the difference was statistically insignificant. Treatment of the hyperlipidemic rats with BC for 4 weeks significantly attenuated the serum concentrations of TG, TC, LDL-c, whereas the serum HDL-c concentration was restored as compared to that in untreated (i.e. HFD alone) rats (Fig. 1a–d). The serum FFA concentration was unchanged (Fig. 1e).

### The effect of BC treatment on body and liver weight

Rats fed HFD for 9 weeks developed hepatomegaly (Table 1). The body weight between HFD-fed and chow diet-fed rats showed no difference (Table 1). After 4-week BC treatment, both body weight and liver/body weight ratio were decreased compared with those untreated (i.e. HFD alone) rats (Table 1). Food intake was identical between HFD and BC-treated groups (Table 1), thus the weight loss upon BC treatment was unrelated to satiety.

**Table 1:** Phenotypic parameters

Items	Chow diet rats (n = 10)	HFD rats (n = 10)	BC treated rats (n = 10)
Body weight (g)-initial	199.43 ± 8.99	204.49 ± 11.33	204.38 ± 13.48
Body weight (g)-final	362.75 ± 22.33	382.71 ± 18.19	356.05 ± 19.72^#^
Liver weight (g)	9.44 ± 1.63	15.10 ± 1.00***	13.50 ± 1.20***
Liver/body weight ratio (%)	2.59 ± 0.36	3.93 ± 0.28***	3.80 ± 0.32***
Daily food intake (g/rat)	20.74 ± 2.01	13.93 ± 2.06***	14.70 ± 3.13***

Data are presented as mean ± SD

***P < 0.001, compared with chow diet group; ^#^ *P* < 0.05, compared with HFD group

### The effect of BC treatment on serum metabolites

Analysis of serum metabolites between HFD-fed (i.e. hyperlipidemia model) and chow diet-fed rats revealed changes in 31 serum metabolites (Additional file 1: Table S1). Of which, metabolites related to fatty acid synthesis or cholesterol synthesis were enhanced, whereas metabolites related to TCA cycle were decreased (Additional file 1: Table S1) upon high fat dieting.

We next determined the effect of BC treatment on serum metabolites in the hyperlipidemic rats. The *R*^*2*^*X* and *Q*^2^ of PCA score analysis were 0.595 and 0.101, respectively; the *R*^2^Y and *Q*^2^ of PLS-DA score analysis were 0.995 and 0.802, respectively. These data indicated the validity of current models.

**Fig. 2:**
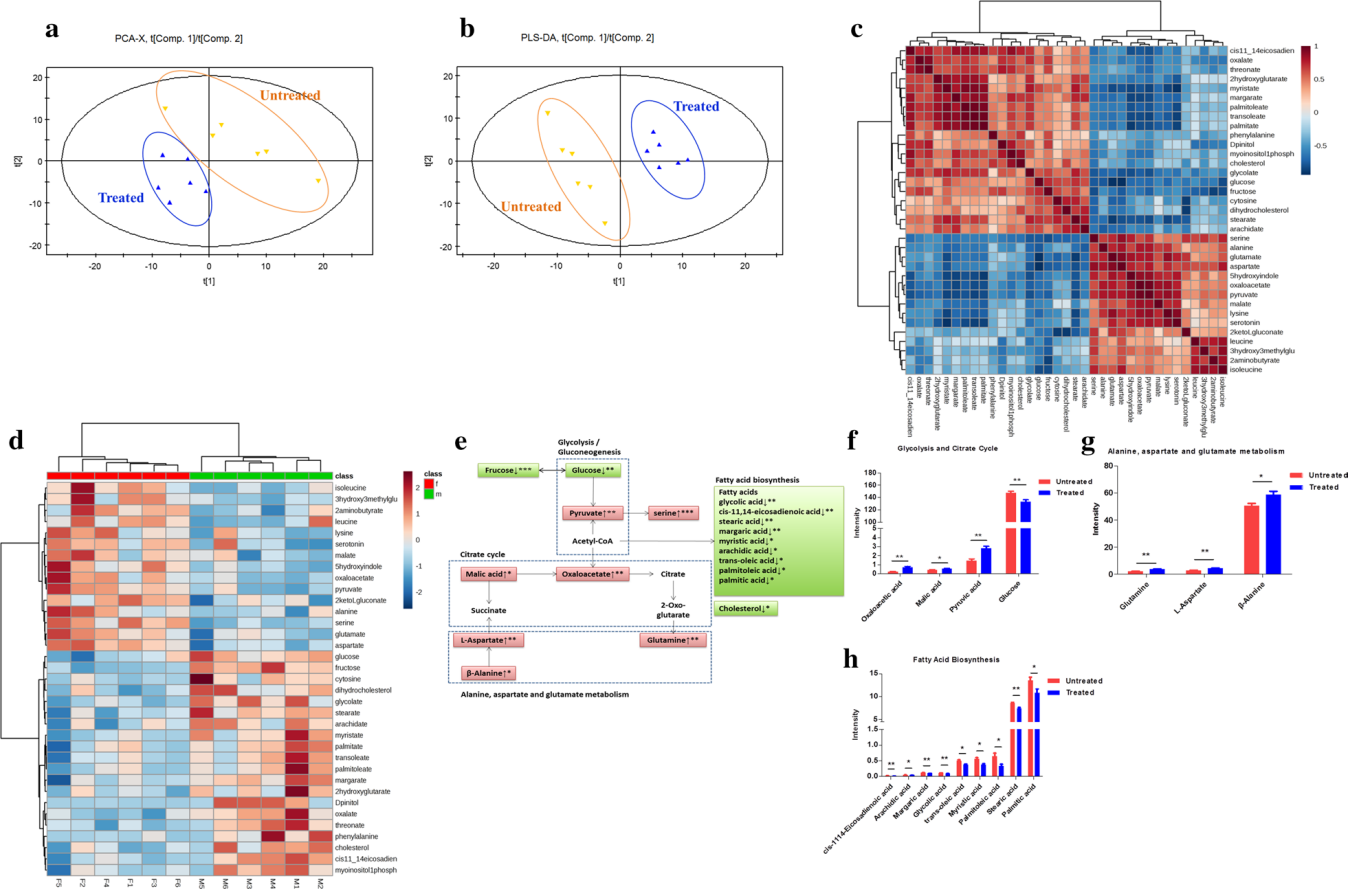
The metabolic profiles in the serum. The HFD induced hyperlipidemic rats were treated with or without BC for 4-week, serum was collected, and metabolomics analysis was made by GC/MS. **a** PCA score plots serum samples from BC treated group and untreated group; **b** scores plots of PLS-DA between untreated group and BC treated group; **c** Pearson’s correlations of the quantities of the 35 metabolites determined from serum samples; **d** heat map showing the fold change (FC) of 19 metabolites.* Shades of green* represent FC decrease while* red* represent FC increase; **e** simplified draft illustrating perturbed pathways involved; **f** differential metabolites between the groups in glycolysis and citrate cycle; **g** differential metabolites between groups in glycolysis and citrate cycle; **h** differential metabolites between the groups in fatty acid biosynthesis. Values were showed as mean peak intensities ± SEM, *P < 0.05, **P < 0.01 and ***P < 0.001, compared with untreated (HFD) group

Distinct clustering of serum metabolites was apparent between control and BC treated groups (Fig. 2a, b). The set of identified metabolites were systematically searched for Pearson’s correlations, and the correlations were indicated in different colors (Fig. 2c). Fold change (FC) of each metabolite was used to construct a heat map (Fig. 2d). Compared with the metabolic profiles of untreated rats, 15 metabolites were increased and 16 decreased in BC treated rats, and the profiles are summarized in Fig. 2e. Among the increased metabolites, oxaloacetic acid, pyruvic acid and malic acid are involved in glycolysis and the TCA cycle (Fig. 2f), glutamic acid, aspartic acid, and alanine are involved in the metabolism of gluconeogenic amino acids (i.e. alanine, aspartate and glutamate) (Fig. 2g). Decreased metabolites, such as* cis*-11,14-eicosadienoic acid, stearic acid, margaric acid, myristic acid, arachidic acid,* trans*-oleic acid, palmitoleic acid, and palmitic acid, are involved in fatty acid biosynthesis (Fig. 2h).

### The effect of BC treatment on urine metabolites

A total of 51 urine metabolites showed difference between HFD-fed and chow diet-fed rats (Additional file 1: Table S2), of which 15 were increased and 41 were decreased. Alterations in these metabolites suggested suppression in the TCA cycle, the pentose phosphate pathway, and purine/pyrimidine metabolism (Additional file 1: Table S2).

**Fig. 3:**
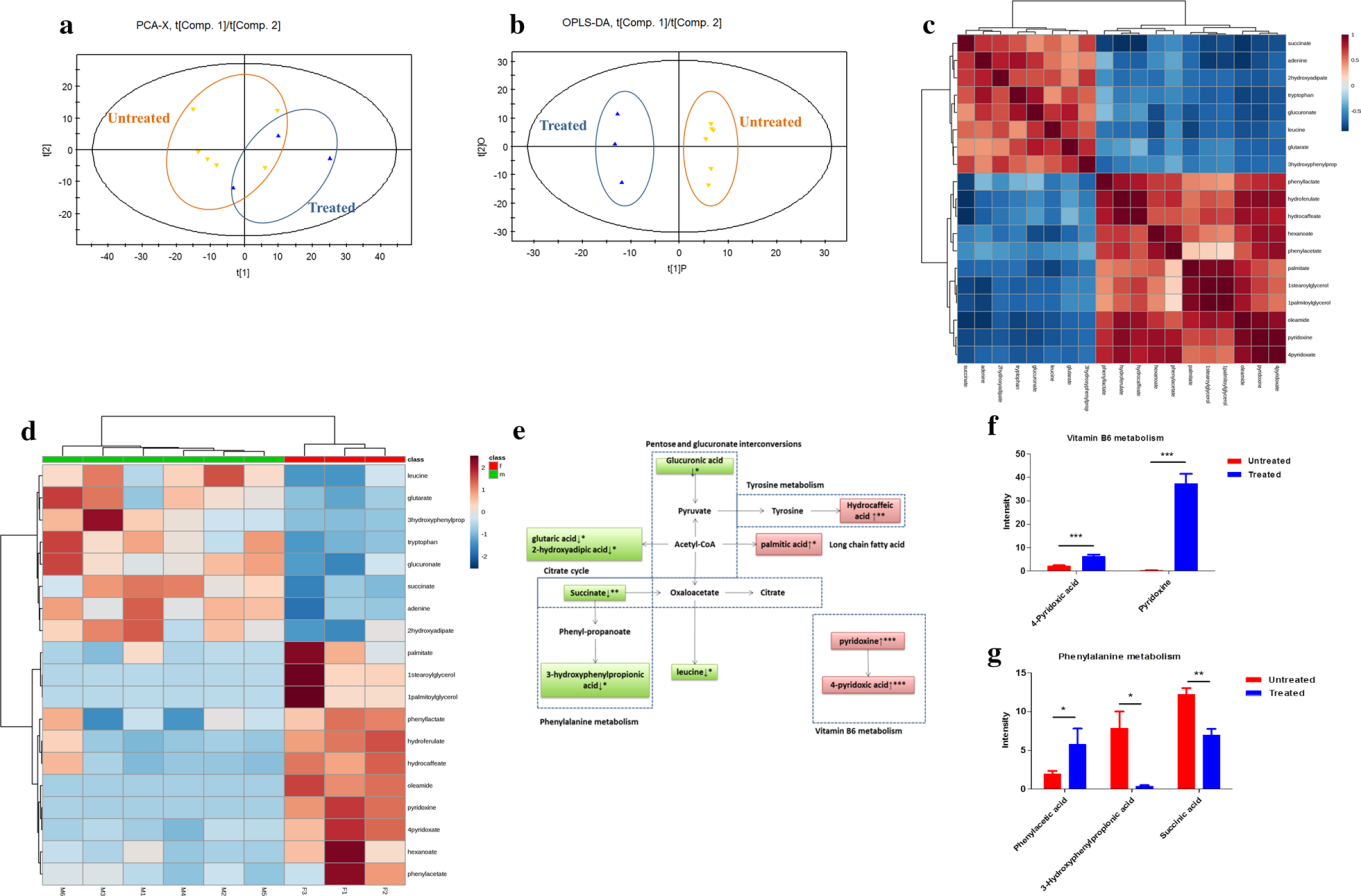
The metabolic profiles in the urine. The HFD induced hyperlipidemic rats were treated with or without BC for 4-week, urine was collected, and metabolomics analysis was made by GC/MS. **a** PCA score plots of urine samples from BC treated group and untreated group; **b** scores plots of OPLS-DA between untreated group and BC treated group; **c** Pearson’s correlations of the quantities of the 35 metabolites determined from rat urine samples; **d** heat map showing the FC of 19 metabolites. *Shades of blue* represent FC decrease while *red* represent FC increase; **e** simplified draft illustrating perturbed pathways involved; **f** differential metabolites between groups in vitamin B_6_ metabolism; **g** differential metabolites between groups in phenylalanine metabolism. Values were showed as mean peak intensities ± SEM. *P < 0.05, **P < 0.01 and ***P < 0.001, compared with untreated (HFD) group

We next determined the effect of BC treatment on urine metabolites in the hyperlipidemic rats. The *R*^2^*X* and *Q*^2^ of PCA score analysis were 0.557 and 0.204, respectively; the *R*^2^Y and *Q*^2^ of OPLS-DA score analysis were 0.995 and 0.824, respectively (Fig. 3a, b), indicating the classifications was well suited for the models, and the BC untreated and treated groups were classified clearly. The set of identified metabolites was systematically searched through for Pearson’s correlations (Fig. 3c), and FC of each metabolite was used to construct a heat map (Fig. 3d). The observed changes in metabolites in the rat urine samples were summarized in Fig. 3e. Notably, metabolites pyridoxine and 4-pyridoxic acid that are related to vitamin B_6_ metabolism were remarkably increased (Fig. 3f) upon BC treatment. In addition, metabolites involved in phenylalanine metabolism, such as succinic acid and 3-hydroxyphenylpropionic acid, were decreased, whereas phenylacetic acid and phenyllactic acid were increased (Fig. 3g).

### The effect of BC treatment on liver metabolites

For liver samples, we identified 36 metabolites in hyperlipidemic rats that distinctly different from those in chow diet group (Additional file 1: Table S3), 16 metabolites were increased and 20 metabolites were decreased in hyperlipidemic rats. These altered metabolites were supposed to be involved in the TCA cycle, the pentose phosphate pathway, fatty acid metabolism, and amino acid metabolism (Additional file 1: Table S3).

**Fig. 4:**
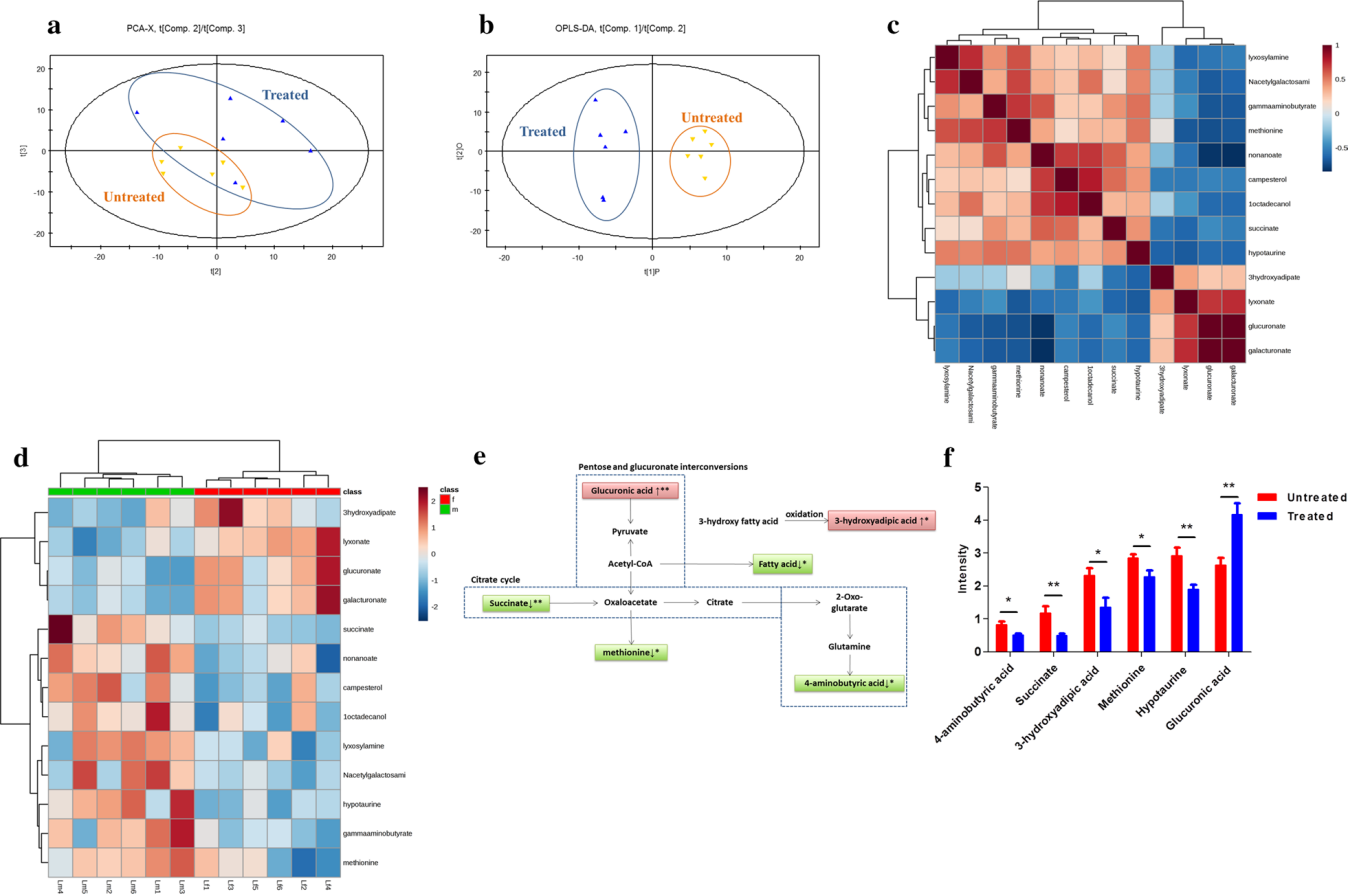
The metabolic profiles in liver tissue. The HFD induced hyperlipidemic rats were treated with or without BC for 4-week, liver tissues were collected, and metabolomics analysis was made by GC/MS. **a** PCA score plots of liver samples from BC treated group and untreated group; **b** scores plots of OPLS-DA between untreated group and BC treated group; **c** Pearson’s correlations of the quantities of the 13 metabolites determined from rat liver samples; **d** heat map showing the FC of 19 metabolites. *Shades of green* represent FC decrease while* red* represent FC increase; **e** simplified draft illustrating perturbed pathways involved; **f** differential metabolites between groups in the liver. Values were showed as mean peak intensities ± SEM. *P < 0.05; **P < 0.01, compared with untreated (HFD) group

We next determined the effect of BC treatment on liver metabolites in the hyperlipidemic rats. The *R*^2^*X* and *Q*^2^ of PCA score analysis were 0.539 and 0.226, respectively; the *R*^2^Y and *Q*^2^ of OPLS-DA score analysis were 0.996 and 0.564, respectively (Fig. 4a, b), validating the classification for the chosen model. The set of identified metabolites was systematically searched through for Pearson’s correlations (Fig. 4c), and FC of each metabolite was used to construct a heat map (Fig. 4d). Metabolites from the BC treated liver samples that were significantly different are summarized in Fig. 4e. Glucuronic acid, lyxonic acid, 3-hydroxyadipic acid and galacturonic acid showed increase, whereas phytosterol, γ-aminobutyric acid, methionine, nonanoic acid, hypotaurine, 1-octadecanol, lyxosylamine and* N*-acetylgalactosamine were decreased (Fig. 4f).

### The effect of BC treatment on fecal metabolites

For the fecal samples, 27 metabolites were up-regulated and 16 metabolites were down-regulated in hyperlipidemic rats (Additional file 1: Table S4). Changes in these metabolites suggested the enhancement in fatty acid and cholesterol synthesis, and the suppression in TCA cycle, tryptophan metabolism, and purine/pyrimidine metabolism (Additional file 1: Table S4).

**Fig. 5:**
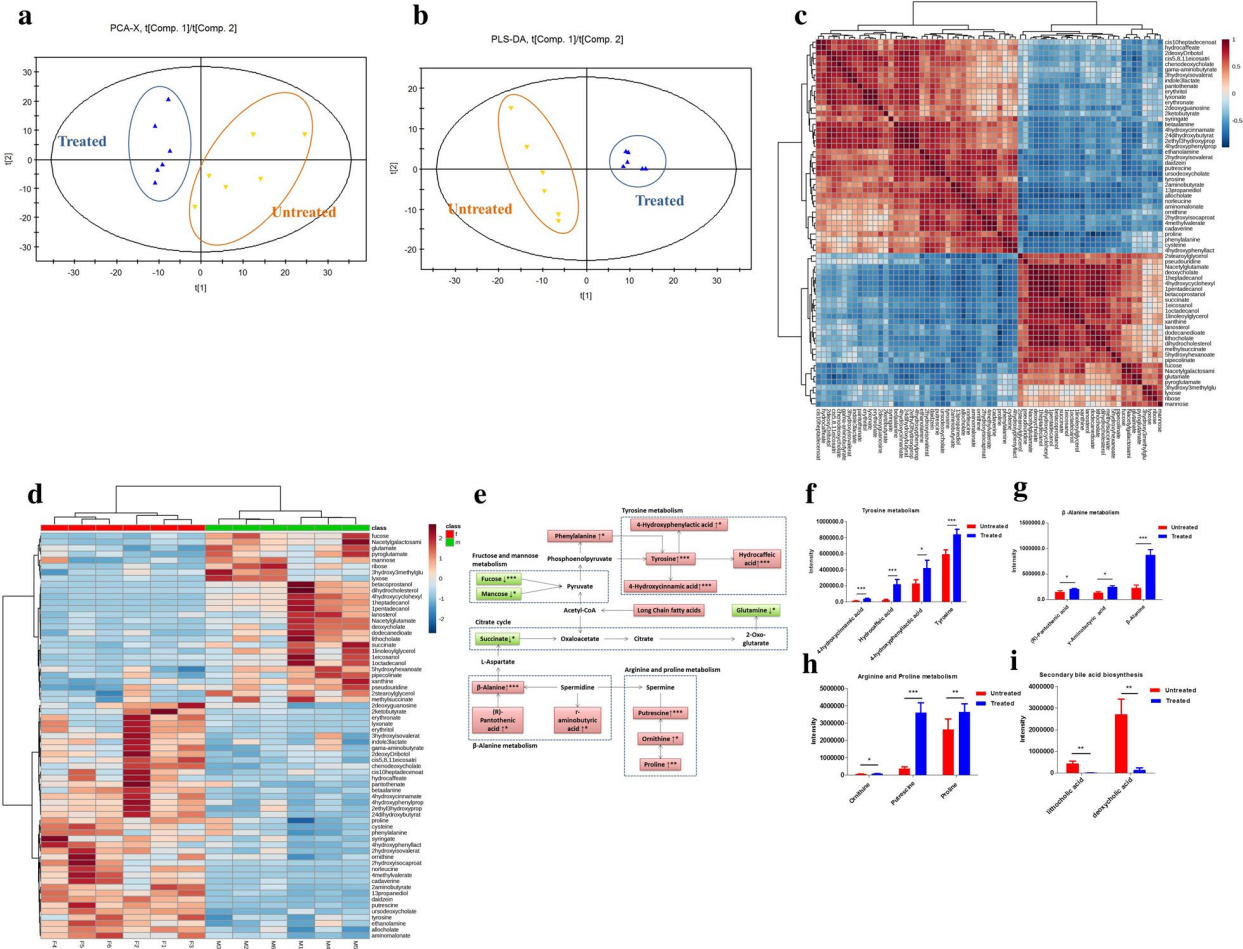
The metabolic profiles in feces. The HFD induced hyperlipidemic rats were treated with or without BC for 4-week, feces were collected, and metabolomics analysis was made by GC/MS. **a** PCA score plots of the feces from BC treated group and untreated group; **b** scores plots of PLS-DA between untreated group and BC treated group; **c** Pearson’s correlations of the quantities of the 67 metabolites determined from the feces samples; **d** heat map showing the FC of 19 metabolites,* shades of green* represent FC decrease while* red* represent FC increase; **e** simplified draft illustrating perturbed pathways involved; **f** differential metabolites between the groups in tyrosine metabolism; **g** differential metabolites between the groups in β-alanine metabolism; **h** differential metabolites between the groups in arginine and proline metabolism; **i** differential metabolites between the groups in secondary bile acid biosynthesis. Values were showed as mean peak intensities ± SEM. *P < 0.05, **P < 0.01 and ***P < 0.001, compared with untreated (HFD) group

We next determined the effect of BC treatment on fecal metabolites in the hyperlipidemic rats. The *R*^2^*X* and *Q*^2^ of PCA score analysis were 0.548 and 0.262, respectively; the *R*^2^Y and *Q*^2^ of PLS-DA score analysis were 0.989 and 0.912, respectively (Fig. 5a, b), indicating the classification was well suited for the models, and the profiles between BC treated rats and hyperlipidemic rats were classified clearly. The set of identified metabolites was systematically searched through for Pearson’s correlations (Fig. 5c). FC of each metabolite was used to construct a heat map (Fig. 5d). The observed changes in endogenous metabolites in rat feces were summarized in Fig. 5e. It was noted that the BC increased the metabolites (tyrosine, hydrocaffeic acid, 4-hydroxycinnamic acid and indole-3-lactic acid) involved in the tyrosine metabolism (Fig. 5f), the metabolites (pantothenic acid, γ-aminobutyric acid and β-alanine) involved in the β-Alanine metabolism (Fig. 5g) and the metabolites (ornithine, proline and putrescine) involved in the arginine and proline metabolism (Fig. 5h), and decreased the metabolites (deoxycholate and lithocholate) involved in the secondary bile acid biosynthesis (Fig. 5i).

### The effect of BC treatment on the structure of microbiota in feces

We used high quality pyrosequencing technology to determine the structure of fecal microbiota. A total of 363,615 usable raw sequences (average of 20,201 sequences per sample) and 34,866 operational taxonomic units (OTUs) were generated by pyrosequencing method from 18 samples. Rarefaction and Shannon diversity curves demonstrated that most of the diversity had already been captured (Additional file 1: Figure S2). The thetayc logarithm showed the gut microbiota could be divided into independent clusters on the basis of the community composition (Additional file 1: Figure S3A, S3B), and the relative abundance of bacterial class also showed clearly distinct (Additional file 1: Figure S3C, S3D). PCoA and NMDS were applied to visualize the similarities or dissimilarities of these data (Additional file 1: Figure S4). The weighted and unweighted unifrac significance test demonstrated that the microbiota structure of the BC treated group was distinct from that of the untreated group (Additional file 1: Table S5).

**Fig. 6:**
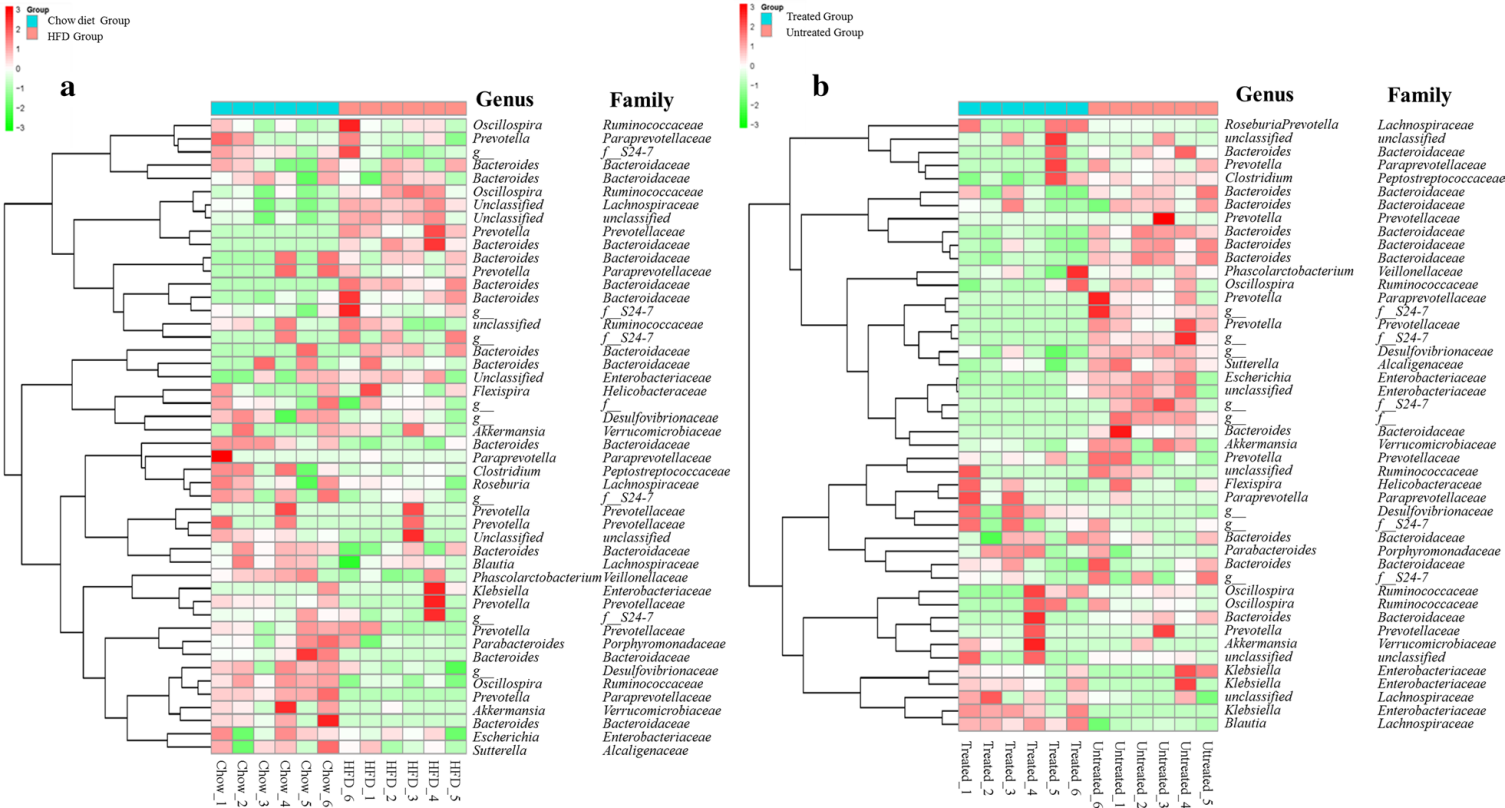
The structure of micorbiota in the feces. Heat map of key OTUs indicating genus-level changes among the groups. The relative abundance of each genus was indicated by a gradient of* color* from* green* (low abundance) to* red* (high abundance). Complete linkage clustering of samples was based on the genus composition and abundance. **a** Chow group versus HFD group; **b** BC treated (HFD) group versus untreated group

We detected 8272 OTUs through the three groups. By plotting the ranked abundance of all 8272 OTUs according to their occurrence, we chose the highest 50 OTUs in species abundance, most of which distributed across such families as *Bacteroidaceae* (12 OTUs), *S24*-*7* (5 OTUs), *Enterobacteriaceae* (5 OTUs), *Prevotellaceae* (5 OTUs), *Ruminococcaceae* (4 OTUs), *Paraprevotellaceae* (4 OTUs), *Lachnospiraceae* (3 OTUs), *Desulfovibrionaceae* (2 OTUs), *Verrucomicrobiaceae* (2 OTUs) and *Lachnospiraceae* (2 OTUs). According to frequency within each sample, the heat map showed the genus level clustering (Fig. 6a), 31 of the 50 identified key OTUs were eliminated or decreased in HFD group, namely *Bacteroides* (3 OTUs), *Prevotella* (4 OTUs) and one OTU to each of the following genera: *Escherichia*, *Sutterella*, *Parabacteroides*, *Clostridium* and *Blautia*. Meanwhile, hyperlipidemic rats showed higher abundance of *Akkermansia*. 27 of the 50 identified key OTUs were eliminated or decreased with BC intervention, whereas the rest of identified OTUs were enriched. The identified OTUs belonging to *Prevotella* (4 OTUs) and one OTU to each of the following genera: *Escherichia*, *Clostridium* and *Sutterella, which* were markedly decreased at genus level in the BC treated group. Meanwhile, higher abundances of *Bacteroides* (10 OTUs), *Parabacteroides* (1 OTU) and *Blautia* (1 OTU) were observed in the BC treated group (Fig. 6b).

### The safety parameters of BC in serum

Analysis of serum ALT and AST showed that HFD-fed rats exhibited increase in these liver enzymes, suggesting compromised liver function was associated with hyperlipidemia (Table 2). However TBIL or TBA in the hyperlipidemic rats were normal (Table 2). Treatment with BC normalized serum AST to control level, and also had a trend in reducing ALT in the hyperlipidemic rats (Table 2). Overall, the BC treatment showed no adverse effect on any of the parameters examined (Table 2), suggesting the dosage of BC was well tolerated in these animals. The food intake and body weight gain were not affected by the BC treatment (Table 1), nor was dry stool or diarrhea observed in BC treated rats (data not shown).

**Table 2:** Serum biochemical parameters related to BC safety

Items	Chow diet rats (n = 10)	HFD rats (n = 10)	BC treated rats (n = 10)
ALT	20.3 ± 2.8	42.2 ± 12.6***	38.0 ± 13.4***
AST	109.2 ± 6.6	151.2 ± 41.1*	107.3 ± 13.4^#^
TBIL	0.7 ± 0.1	0.6 ± 0.5	0.6 ± 0.2
TBA	4.5 ± 1.4	5.4 ± 1.9	7.1 ± 3.4

Data are presented as Mean ± SD

* *P* < 0.05, *** *P* < 0.001 compared with chow diet group; ^#^ *P* < 0.05, compared with HFD group

## Discussion

Hyperlipidemia is one of the components of metabolic syndrome, the pathophysiology is very complex and has been only partially elucidated. Insulin resistance and obesity are implicated in the development of hyperlipidemia, however, there is debate regarding whether they are the cause or the consequences of a more far-reaching metabolic derangement. It is generally accepted that the current food environment is the contributor. For the treatment of hyperlipidemia, dietary modification is the initial approach, but many patients require pharmacological therapy to reduce cardiovascular risk. The most commonly used agents are statins (3-hydroxy-3-methyl glutaryl coenzyme A reductase inhibitors) and fibrates (peroxisome proliferator-activated receptor-alpha agonists), these agents, while effective, cause a markedly increased risk of myopathy and rhabdomyolysis [5].

The ingredients of BC were all derived from natural plants and dietary supplements, unless overdose, the safety can be guaranteed. BBR is the main ingredient of BC, and many studies have shown the multiple molecular mechanisms and pathways underlying the beneficial effects of BBR in metabolic diseases. Main targets of BBR on lipid and glucose metabolism are low-density lipoprotein receptor (LDLR) and the insulin receptor (InsR), and this one-drug-multiple-target characteristic is required for the treatment of hyperlipidemia. We added oryzanol and vitamin B_6_ at a dose much lower than the tolerable upper intake level, and studies have confirmed their benefits on metabolic diseases, however, with a much higher dosage (oryzanol at 3.2 g/kg, vitamin B_6_ at 50 mg/kg) [13, 24]. The current study confirmed the significant lipid-lowering effect of BC and did not detect any evidence for side effects with the 4-week BC intervention. These results were consistent with the data obtained in BBR studies [25].

By applying integrated metabolomics in serum, urine, liver and feces, we identified a number of metabolites in the hyperlipidemic rats treated with BC. The most obvious finding of our GC/MS analysis was the metabolites related to lipid metabolism. Blood lipids, derived from food intake or adipose tissue and liver, are mainly fatty acid derivatives and cholesterol [26]. Besides biochemistry evidence of lipid-lowering activity, BC also affected the lipid metabolism related metabolites. FFAs are catabolized in the mitochondria to produce energy, and the process generated numerous metabolites [27]. The change of serum metabolites suggested an attenuated fatty acids synthesis pathway and an enhanced β-oxidation pathway in BC treated hyperlipidemic rats.

Putrescine is a polyamine that is important for cell functions, polyamine can participate in fatty acid metabolism by spermidine/spermine *N*^1^-acetyltransferase (SSAT), and enhanced ornithine decarboxylase (ODC) and SSAT activities may promote acetyl-CoA conversion from fatty acid synthesis to acetylation of polyamines. In other words, increase of putrescine indicates enhanced fatty acid β-oxidation [28].

Another important finding was the metabolites related to glycolysis and the TCA cycle. HFD-induced hyperlipidemic rats demonstrated glycolysis inhibition and energy metabolism dysfunction, and BC were found to restore this status by up-regulating the level of glucose, pyruvate, glucose-6-phosphate, oxaloacetic acid and succinate among others. Pyruvate, a key metabolite linking glycolysis to the TCA cycle, are derived from glycolysis and can be used to produce acetyl-CoA, then enters into the TCA cycle [17]. Hyperlipidemia generates large amount of acetyl-CoA, which would inhibit pyruvate conversion in a negative feedback way [29]. Our results are in line with previous study that shows similar alterations in glucose and energy metabolism [30], indicating BC treatment affected glycolysis process and the TCA cycle by recovering the suppressed energy metabolism. However, the scope of this study was limited in terms of glucose metabolism, since blood glucose levels did not increase in response to the dietary, thus the effect of BC treatment on hyperglycemia was unable to determine.

The levels of the gluconeogenic amino acids (e.g. alanine, valine, threonine, glutamine, serine, glycine, aspartate, proline) as well as those of the ketogenic amino acids (e.g. isoleucine, lysine, tyrosine, phenylalanine, tryptophan) were increased upon BC treatment. Phenylalanine and tyrosine are the precursor of epinephrine, and tyrosine consumption increases when epinephrine is in demand for lipid metabolism [31]. BC administration caused tyrosine level decrease in our study, implicating that the epinephrine pathway might be involved. The glutathione precursor, glutamate is the first line of defense against free radicals in the liver, and is an essential amino acid during the pathogenesis of metabolic diseases [32]. Although the increase of hepatic glutamate in our study was not statistically significant, the glutamate level in both urine and feces markedly decreased, indicating the reduced degradation and secretion of glutamate. This finding demonstrated that anti-oxidative activity is one of the possible properties of the BC, and it seemed to be in agreement of the results obtained from gallic acid studies [33]. The branched chain amino acids (BCAAs: leucine, isoleucine, and valine) play important roles in promoting protein synthesis [34], glucose metabolism and oxidation [35], and regulating leptin secretion [36]. High levels of BCAAs contribute to obesity-related insulin resistance and glucose intolerance [37], and a recent study has reported that the blood concentration of five branched-chain and aromatic amino acids (isoleucine, leucine, valine, tyrosine, and phenylalanine) could predict the risk of future diabetes [38]. BC were found to have certain actions on the metabolites of BCAAs, which might be contribute to ketogenesis suppression.

Vitamin B_6_ consists of pyridoxal, pyridoxine, and pyridoxamine, which functions as essential cofactors for enzymes involved in various metabolic activities [39]. The biologically active form is the phosphate ester derivative pyridoxal 5′-phosphate (PLP), which could reflect long-term body storage [40]. Low plasma PLP is the marker of diminished vitamin B_6_ that adversely affects polyunsaturated fatty acid metabolism, arachidonic acid and hepatic cholesterol biosynthesis. An increase in pyridoxine and 4-pyridoxic acid was present in BC treated rats, suggesting an enhanced vitamin B_6_ metabolism.

Interestingly, the production of fecal secondary bile acids, deoxycholate (DCA) and lithocholate (LCA), which are synthesized from primary bile acids cholate and chenodeoxycholate by a few species of intestinal bacteria, respectively [41], were markedly reduced in BC treatment group. LCA, the most toxic bile acid [42], could induce DNA damage and inhibit DNA repair enzymes [43]. Recently, it has been reported that dietary vitamin B_6_ could reduce the production of LCA in HFD rats. These findings implied vitamin B_6_ may be beneficial for intestine health under HFD conditions [44].

Recent studies have shown the diversity and composition of gut microbiome altered in metabolic diseases. *Bacteroides* is negatively correlated with energy intake and obesity [45]. *Blautia* is an acetogen belongs to the known SCFA-producing bacteria. Abundance of the SCFA producer helps to alleviate systemic inflammation by promoting energy intake and elevating intestinal SCFA levels. Our study suggests that structural modulation of gut microbiota was in response to BC intervention, and these changes might contribute to the enhancement of energy metabolism.

As mentioned, BBR causes side effects related to its impact on bowel motility including abdominal pain, distention, nausea, vomiting, and constipation [46]. During the process of our experiment, no obvious gastrointestinal symptom was observed in the BC treated rats. The significant increase in serotonin and putrescine are intriguing. Serotonin mediates effects not only on sleep, emotion and appetite but also on neural reflexes that regulate gastrointestinal motility, secretion, and sensation [47]. Putrescine, synthesized from ornithine by ODC, may be an important substance with respect to intestinal function as it is an instant energy source in times of energy deficiency in gut metabolism [48]. The increases in serotonin and putrescine may therefore imply BC could reduce gastrointestinal discomfort discovered in previous BBR research by regulating colonic motor activity.

## Conclusions

The present study determined the anti-hyperlipidemia effect of BC on diet-induced hyperlipidemic rats. According to the alterations in metabolites from different types of sample and gut microbiota structure, BC were inferred to partially recover both metabolism dysfunction and intestinal environment through several metabolic pathways (glycolysis and TCA cycle, β-oxidation of fatty acids, synthesis of fatty acid and cholesterol, amino acid metabolism, vitamin B_6_ metabolism, and secondary bile acid biosynthesis) and modulation of the micorbiota. What’s more, these findings imply the possibility of BC treatment on improving the gastrointestinal discomfort. This research provided a framework for the exploration of natural products on metabolic diseases in holistic and systematic manners. Further researches are required to optimize the specialized targets of BC and establish a comprehensive screening system for hyperlipidemia.

## Additional file

10.1186/s12967-016-0987-5 Changes of serum parameters at the 4th week of feeding in rats. Male Wistar rats (8 weeks of age) placed on HFD or chow diet for 4 weeks, blood samples were collected randomly from tail vein of 6 rats, and serum was separated, the lipid profiles (TC, TG, HDL-c and LDL-c) were analyzed. The data were presented as the mean ± SD. ***P* < 0.01, compared with chow group. **Figure S2.** Evaluation of the sequencing depth in samples from the chow diet group, HFD group and BC treated group in the pyrosequencing run. **Figure S3.** Similarity of community on the basis of thetayc logarithm among the chow diet, HFD and BC treated groups. **Figure S4.** PCoA and NMDS plot of chow diet group, HFD group and BC treated group. **Table S1.** Endogenous metabolites of the serum from chow diet group and HFD group. **Table S2.** Endogenous metabolites of the urine from chow diet group and HFD group. **Table S3.** Endogenous metabolites of the liver from chow diet group and HFD group. **Table S4.** Endogenous metabolites of the feces from chow diet group and HFD group. **Table S5.** The weighted and unweighted unifrac significance test.

## Abbreviations

ALT: alanine aminotransferase
ANOVA: analysis of variance
AST: aspartate aminotransferase
BBR: berberine
BC: berberine compounds
BCAA: branched chain amino acid
BSTFA: *N*,O-bis(trimethylsilyl)-trifluoroacetamide
CMC-Na: sodium carboxy methylcellulose
DCA: deoxycholate
FC: fold change
FFAs: free fatty acids
GC/MS: gas chromatography/mass spectrometry
HDL-c: high-density lipoprotein cholesterol
HFD: high fat diet
InsR: insulin receptor
LCA: lithocholate
LDL-c: low-density lipoprotein cholesterol
LDLR: low-density lipoprotein receptor
MSR: MiSeq reporter software
NMDS: nonmetric multidimensional scaling
ODC: ornithine decarboxylase
OPLS-DA: orthogonal partial least squares-discriminant analysis
OTU: operational taxonomic unit
PCA: principal component analysis
PCoA: principal coordinate analysis
PLP: pyridoxal 5′-phosphate
PLS-DA: partial least squares-discriminant analysis
RDP: ribosomal database project
RSB: resuspension buffer
SSAT: spermidine/spermine-*N*^1^-acetyltransferase
TBA: total bile acid
TBIL: total bilirubin
TC: total cholesterol
TG: triglycerides
TMCS: trimethylchlorosflane
