# Supplementary material for: Integrative analysis of metabolome and gut microbiota in diet-induced hyperlipidemic rats treated with berberine compounds
Source: J Transl Med. 2016 Aug 5;14:237. doi: 10.1186/s12967-016-0987-5 (PMC4975912; doi:10.1186/s12967-016-0987-5)
Supplement: Supplementary file 1 — 10.1186/s12967-016-0987-5 Changes of serum parameters at the 4th week of feeding in rats. Male Wistar rats (8 weeks of age) placed on HFD or chow diet for 4 weeks, blood samples were collected randomly from tail vein of 6 rats, and serum was separated, the lipid profiles (TC, TG, HDL-c and LDL-c) were analyzed. The data were presented as the mean ± SD. **P < 0.01, compared with chow group. Figure S2. Evaluation of the sequencing depth in samples from the chow diet group, HFD group and BC treated group in the pyrosequencing run. Figure S3. Similarity of community on the basis of thetayc logarithm among the chow diet, HFD and BC treated groups. Figure S4. PCoA and NMDS plot of chow diet group, HFD group and BC treated group. Table S1. Endogenous metabolites of the serum from chow diet group and HFD group. Table S2. Endogenous metabolites of the urine from chow diet group and HFD group. Table S3. Endogenous metabolites of the liver from chow diet group and HFD group. Table S4. Endogenous metabolites of the feces from chow diet group and HFD group. Table S5. The weighted and unweighted unifrac significance test. [file 12967_2016_987_MOESM1_ESM.docx]

**Figure S1.** Changes of serum parameters at the 4^th^ week of feeding in rats. Male Wistar rats (8 weeks of age) placed on HFD or chow diet for 4 weeks, blood samples were collected randomly from tail vein of 6 rats, and serum was separated, the lipid profiles (TC, TG, HDL-c and LDL-c) were analyzed. The data were presented as the mean ± SD. ^**^*P*<0.01, compared with chow group.


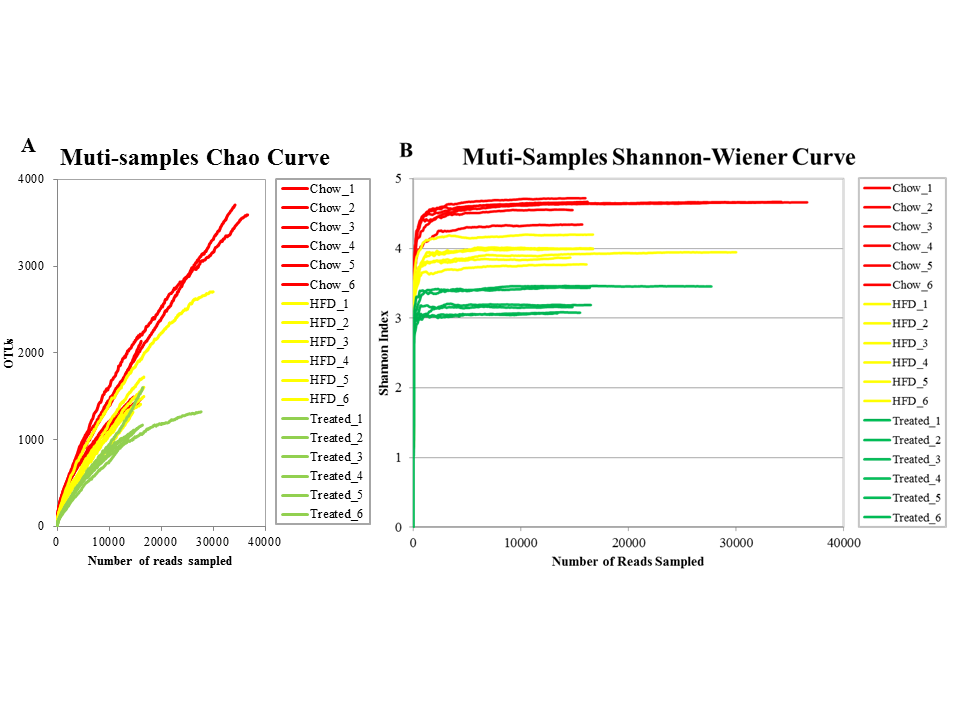


**Figure S2.** Evaluation of the sequencing depth in samples from the chow diet group, HFD group and BC treated group in the pyrosequencing run. **A** Rarefaction curves of sequencing samples; **B** Shannon diversity index curves of sequencing samples.


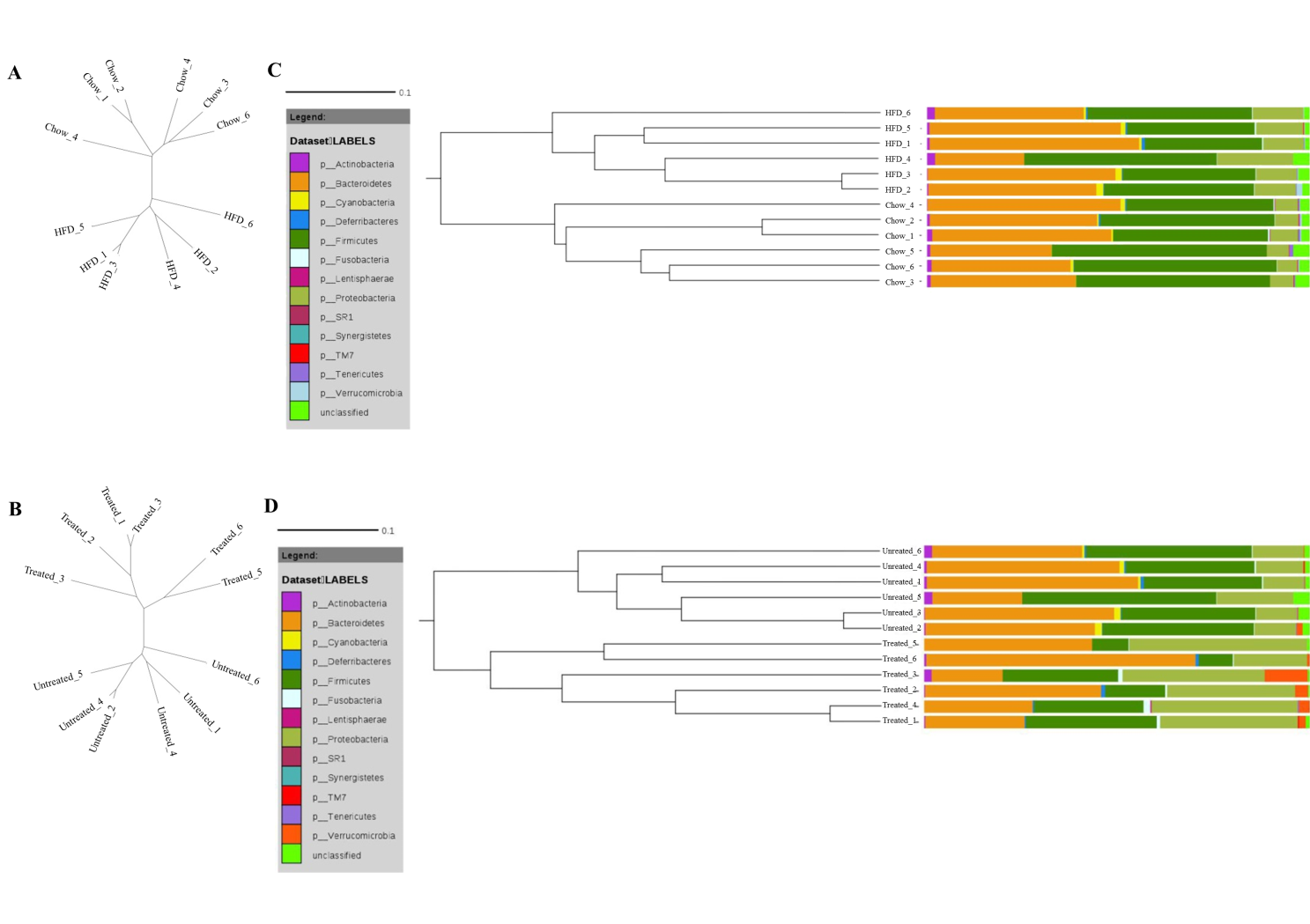


**Figure S3.** Similarity of community on the basis of thetayc lgorithm among the chow diet, HFD and BC treated groups. **A** Differentiation in bacterial communities from chow diet group and HFD group; **B** Differentiation in bacterial communities from untreated group and treated group. Community differentiation was measured by using the thetayc lgorithm; the scale bar indicated the distance between clusters in thetayc units; **C** The relative abundance per subject of microbiota V3 OTUs from chow diet group and HFD group based on the thetayc lgorithm; **D** The relative abundance per subject of microbiota V3 OTUs from treated and untreated group based on the thetayc lgorithm. The relative abundance expressed as the percentage of all V3 tags from that subject. In the legend, the 14 most abundant microbiota OUTs were listed at phylum level.


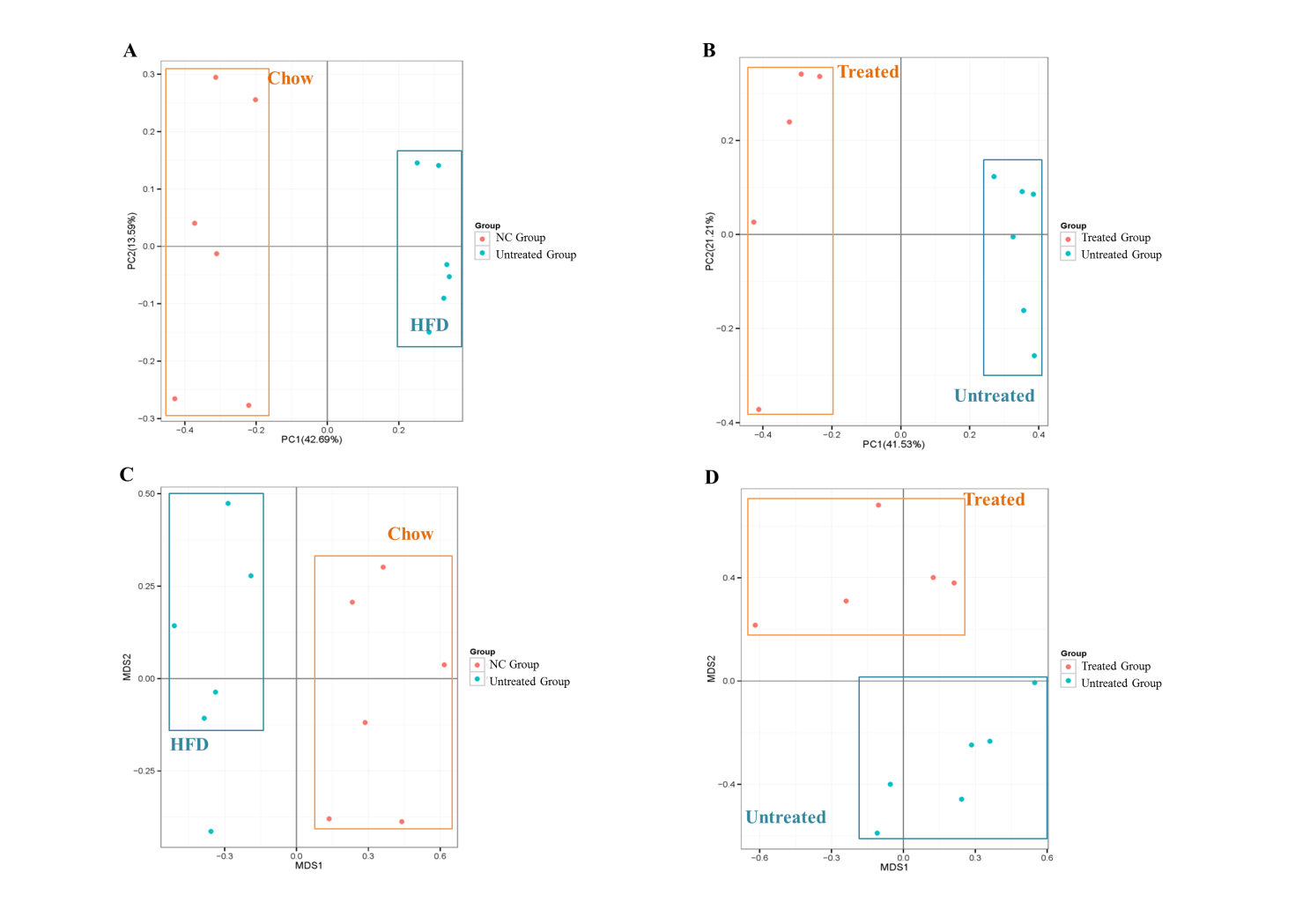


**Figure S4.** PCoA and NMDS plots among groups. **A** PCoA plot of chow diet group and HFD group; **B** PCoA plot of treated group and untreated group; **C** NMDS plot of chow diet group and HFD group; **D** NMDS plot of treated group and untreated group. Samples were grouped by different colors and the percent of variation was explained by each axis. The closer the distance of samples was, the more similar the sample composition.

**TABLE S1.** Endogenous metabolites of the serum from normal chow diet group and HFD group

| **Metabolites** | **Formula** | **Mol Weight** | **VIP** | **p-value^a^** | **FC** | **HMDB** | **KEGG** | **Pathway(KEGG)** |
| --- | --- | --- | --- | --- | --- | --- | --- | --- |
| **oxaloacetic acid** | C_4_H_4_O_5_ | 132.0716 | 1.69 | 8.90E-03 | ↑ | HMDB00223 | C00036 | Glycolysis/Gluconeogenesis;  Citrate cycle (TCA cycle) |
| **glucose** | C_6_H_12_O_6_ | 180.1559 | 1.73 | 6.94E-03 | ↑ | HMDB00122 | C00031 | Glycolysis / Gluconeogenesis;  Pentose phosphate pathway |
| **pyruvic acid** | C_3_H_4_O_3_ | 88.0621 | 1.58 | 1.77E-02 | ↑ | HMDB00243 | C00022 | Glycolysis/Gluconeogenesis;  Citrate cycle(TCA cycle);  Pentose phosphate pathway |
| **citric acid** | C_6_H_8_O_7_ | 192.1235 | 1.57 | 2.06E-02 | ↑ | HMDB00094 | C00158 | Citrate cycle (TCA cycle);  Alanine, aspartate and glutamate metabolism |
| **succinic acid** | C_4_H_6_O_4_ | 118.088 | 1.92 | 1.70E-03 | ↓ | HMDB00254 | C00042 | Citrate cycle (TCA cycle);  Alanine,aspartate and glutamate metabolism |
| **xylulose** | C_5_H_10_O_5_ | 150.1299 | 1.89 | 1.71E-03 | ↑ | HMDB01644 | C00310 | Pentose and glucuronate interconversions |
| **gluconic acid** | C_6_H_12_O_7_ | 196.1553 | 2.09 | 1.30E-04 | ↓ | HMDB00625 | C00257 | Pentose phosphate pathway |
| **glyceric acid** | C3H6O4 | 106.0773 | 1.55 | 2.02E-02 | ↓ | HMDB00139 | C00258 | Pentose phosphate pathway;  Glycine,serine and threonine metabolism;  Glycerolipid metabolism |
| **threonine** | C_3_H_7_NO_2_ | 89.0932 | 2.16 | 2.74E-05 | ↓ | HMDB00167 | C00188 | Glycine,serine and threonine metabolism |
| **serine** | C_2_H_5_NO_2_ | 75.0666 | 2.26 | 6.81E-07 | ↓ | HMDB00187 | C00065 | Glycine,serine and threonine metabolism;  Cysteine and methionine metabolism |
| **valine** | C_5_H_11_NO_2_ | 117.1463 | 1.61 | 1.45E-02 | ↓ | HMDB00883 | C00183 | Valine, leucine and isoleucine degradation |
| **3-methyl-2-ketovaleric acid** |  |  | 1.92 | 1.34E-03 | ↑ | HMDB00491 | C03465 | Valine, leucine and isoleucine degradation |
| **2-ketoisovaleric acid** | C_5_H_8_O_3_ | 116.1152 | 1.79 | 4.71E-03 | ↑ | HMDB00019 | C00141 | Valine,leucine and isoleucine degradation |
| **isoleucine** | C_6_H_13_NO_2_ | 131.1729 | 1.69 | 8.67E-03 | ↓ | HMDB00172 | C00407 | Valine,leucine and isoleucine degradation |
| **oleic acid** | C_18_H_34_O_2_ | 282.4614 | 1.81 | 3.61E-03 | ↓ | HMDB00207 | C00712 | Long chain fatty acid |
| **arachidonic acid** | C_20_H_32_O_2_ | 304.4669 | 1.70 | 7.91E-03 | ↑ | HMDB01043 | C00219 | Long chain fatty acid |
| **cis-11,14-Eicosadienoic acid** | C_20_H_36_O_2_ | 308.4986 | 1.67 | 1.00E-02 | ↓ | HMDB05060 | C16525 | Long chain fatty acid |
| **alpha-Linolenic acid** | C_18_H_30_O_2_ | 278.4296 | 1.66 | 1.05E-02 | ↑ | HMDB01388 | C06427 | Long chain fatty acid |
| **2-hydroxybutyric acid** | C_4_H_8_O_3_ | 104.1045 | 1.70 | 8.62E-03 | ↑ | HMDB00008 | C05984 | fatty acid,monohydroxy |
| **1-stearoylglycerol** | C_21_H_42_O_4_ | 358.5558 | 1.41 | 4.04E-02 | ↑ | HMDB31075 |  | lipid |
| **aspartic acid** | C_4_H_7_NO_4_ | 133.1027 | 1.87 | 2.12E-03 | ↓ | HMDB00191 | C00049 | Alanine,aspartate and glutamate metabolism; Glycine,serine and threonine metabolism;  Cysteine and methionine metabolism |
| **ornithine** | C_5_H_12_N_2_O_2_ | 132.161 | 1.83 | 2.99E-03 | ↓ | HMDB00214 | C00077 | Arginine and proline metabolism |
| **pipecolinic acid** | C_6_H_11_NO_2_ | 129.157 | 1.55 | 2.13E-02 | ↑ | HMDB00070 | C00408 | Lysine degradation |
| **oxalic acid** | C_2_H_2_O_4_ | 90.0349 | 1.75 | 5.74E-03 | ↓ | HMDB02329 | C00209 | Microbial metabolism in diverse environments; Chloroalkane and chloroalkene degradation; Glyoxylate and dicarboxylate metabolism |
| **ethanolamine** | C_2_H_7_NO | 61.0831 | 2.02 | 3.73E-04 | ↓ | HMDB00149 | C00189 | Phosphonate and phosphinate metabolism;  Glycerophospholipid metabolism |
| **myo-inositol-1-phosphate** | C_6_H_13_O_9_P | 260.1358 | 2.02 | 3.72E-04 | ↑ | HMDB00213 | C04006 | Inositol phosphate metabolism |
| **campesterol** | C_28_H_48_O | 400.6801 | 1.92 | 1.56E-03 | ↑ | HMDB02869 | C01789 | A dietary phytosterol found in plants, lowering cholesterol |
| **4-deoxyerythronic acid** | C_4_H_8_O_4_ | 120.1039 | 1.84 | 2.74E-03 | ↓ | HMDB00498 | — | — |
| **2-Keto-l-gluconic acid** | C_6_H_10_O_7_ | 194.1394 | 1.80 | 3.98E-03 | ↓ | HMDB11732 | C15673 | — |
| **indole-3-propionic acid** | C_11_H_11_NO_2_ | 189.2105 | 1.67 | 1.20E-02 | ↑ | HMDB02302 | — | — |
| **3-hydroxy-3-methylglutaric acid** | C_6_H_10_O_5_ | 162.1406 | 1.44 | 3.82E-02 | ↑ | HMDB00355 | C03761 | — |

Note: ^a^Comparison of differential metabolites between chow diet group and HFD group with a Student’s *t* test; FC: fold change. VIP was obtained from PLS-DA with a threshold of 1.0. The up and down arrows indicate a respective increased or decreased concentration of each metabolite in the chow diet group versus HFD group (chow diet / HFD).

**TABLE S2.** Endogenous metabolites of the urine from normal chow diet group and HFD group

| **Metabolites** | **Formula** | **Mol Weight** | **VIP** | **p-value** | **FC** | **HMDB** | **KEGG** | **Pathway(KEGG)** |
| --- | --- | --- | --- | --- | --- | --- | --- | --- |
| **pyruvic acid** | C_3_H_4_O_3_ | 88.0621 | 1.24 | 4.46E-02 | ↓ | HMDB00243 | C00022 | Glycolysis |
| **gluconic acid** | C_6_H_12_O_7_ | 196.1553 | 1.30 | 1.10E-02 | ↓ | HMDB00625 | C00257 | Pentose phosphate pathway |
| **ribose** | C_5_H_10_O_5_ | 150.1299 | 1.23 | 1.56E-02 | ↓ | HMDB00283 | C00121 | Pentose phosphate pathway |
| **glyceric acid** | C_3_H_6_O_4_ | 106.0773 | 1.09 | 3.88E-02 | ↓ | HMDB00139 | C00258 | Pentose phosphate pathway;  Glycine,serine and threonine metabolism |
| **citric acid** | C_6_H_8_O_7_ | 192.1235 | 1.13 | 3.70E-02 | ↓ | HMDB00094 | C00158 | Citrate cycle (TCA cycle) |
| **succinic acid** | C_4_H_6_O_4_ | 118.088 | 1.29 | 3.45E-02 | ↓ | HMDB00254 | C00042 | Citrate cycle (TCA cycle);  Phenylalanine metabolism |
| **creatinine** | C_4_H_7_N_3_O | 113.1179 | 1.16 | 2.59E-02 | ↑ | HMDB00562 | C00791 | Arginine and proline metabolism |
| **pyrrole-2-carboxylic acid** | C_5_H_5_NO_2_ | 111.0987 | 1.07 | 4.78E-02 | ↓ | HMDB04230 | C05942 | Arginine and proline metabolism |
| **dehydroascorbic acid** | — | — | 1.72 | 1.11E-02 | ↓ | HMDB01264 | C00425 | Ascorbate and aldarate metabolism |
| **threonic acid** | C_4_H_8_O_5_ | 136.1033 | 1.35 | 1.64E-02 | ↓ | HMDB00943 | C01620 | Ascorbate and aldarate metabolism |
| **3-hydroxybenzoic acid** | C_7_H_6_O_3_ | 138.1207 | 1.36 | 1.45E-02 | ↑ | HMDB02466 | C00587 | Benzoate degradation |
| **pantothenic acid** | C_9_H_17_NO_5_ | 219.235 | 1.14 | 4.07E-02 | ↓ | HMDB00210 | C00864 | beta-Alanine metabolism;  Pantothenate and CoA biosynthesis |
| **uracil** | C_4_H_4_N_2_O_2_ | 112.0868 | 1.10 | 4.16E-02 | ↓ | HMDB00300 | C00106 | beta-Alanine metabolism;  Pyrimidine metabolism |
| **3-hydroxypropionic acid** | C_3_H_6_O_3_ | 90.0779 | 1.60 | 2.32E-04 | ↓ | HMDB00700 | C01013 | beta-Alanine metabolism;  Propanoate metabolism |
| **4-hydroxybutyric acid** | C_4_H_8_O_3_ | 104.1045 | 1.38 | 7.12E-03 | ↓ | HMDB00710 | C00989 | Butanoate metabolism |
| **alanine** | C_3_H_7_NO_2_ | 89.0932 | 1.39 | 6.54E-03 | ↓ | METPA0179 | C01401 | Alanine, aspartate and glutamate metabolism;  Cyanoamino acid metabolism |
| **cysteine** | — | — | 1.37 | 4.82E-03 | ↓ | METPA0075 | C00736 | Cysteine and methionine metabolism |
| **sarcosine** | C_3_H_7_NO_2_ | 89.0932 | 1.55 | 6.32E-03 | ↑ | HMDB00271 | C00213 | Glycine, serine and threonine metabolism |
| **glycine** | C_2_H_5_NO_2_ | 75.0666 | 1.33 | 2.68E-02 | ↑ | HMDB00123 | C00037 | Glycine, serine and threonine metabolism;  Cyanoamino acid metabolism |
| **palmitic acid** | C_16_H_32_O2 | 256.4241 | 1.22 | 1.69E-02 | ↑ | HMDB00220 | C00249 | Long chain fatty acid |
| **stearic acid** | C_18_H_36_O2 | 284.4772 | 1.31 | 8.59E-03 | ↑ | HMDB00827 | C01530 | Long chain fatty acid |
| **glutaric acid** | C_5_H_8_O_4_ | 132.1146 | 1.33 | 6.97E-03 | ↓ | HMDB00661 | C00489 | Lysine degradation |
| **nicotinic acid** | C_6_H_5_NO_2_ | 123.1094 | 1.14 | 2.96E-02 | ↓ | HMDB01488 | C00253 | Nicotinate and nicotinamide metabolism |
| **arabitol** | C_5_H_12_O_5_ | 152.1458 | 1.31 | 1.12E-02 | ↓ | HMDB00568 | C01904 | Pentose and glucuronate interconversions |
| **benzoic acid** | C_7_H_6_O_2_ | 122.1213 | 1.69 | 3.73E-03 | ↑ | HMDB01870 | C00180 | Phenylalanine metabolism |
| **3-hydroxyphenylacetic acid** | C_8_H_8_O_3_ | 152.1473 | 1.83 | 4.57E-05 | ↑ | HMDB00440 | C05593 | Phenylalanine metabolism |
| **indole-3-lactic acid** | C_11_H_11_NO_3_ | 205.2099 | 1.22 | 2.01E-02 | ↓ | HMDB00671 | C02043 | Tryptophan metabolism |
| **5-hydroxyindoleacetic acid** | C_10_H_9_NO_3_ | 191.1834 | 1.52 | 2.46E-03 | ↓ | HMDB00763 | C05635 | Tryptophan metabolism |
| **leucine** | C_6_H_13_NO_2_ | 131.1729 | 1.39 | 3.75E-03 | ↓ | HMDB00687 | C00123 | Valine, leucine and isoleucine degradation |
| **2-ketoisocaproic acid** | C_6_H_10_O_3_ | 130.1418 | 1.30 | 1.04E-02 | ↓ | HMDB00695 | C00233 | Valine, leucine and isoleucine degradation |
| **methylmalonic acid** | C_4_H_6_O_4_ | 118.088 | 1.12 | 4.23E-02 | ↓ | HMDB00202 | C02170 | Propanoate metabolism;  Valine,leucine and isoleucine degradation |
| **adenine** | C_5_H_5_N_5_ | 135.1267 | 1.30 | 1.57E-02 | ↓ | HMDB00034 | C00147 | Purine metabolism |
| **uric acid** | C_5_H_4_N_4_O3 | 168.1103 | 1.25 | 1.40E-02 | ↑ | HMDB00289 | C00366 | Purine metabolism |
| **orotic acid** | C_5_H_4_N_2_O_4_ | 156.0963 | 1.15 | 4.26E-02 | ↓ | HMDB00226 | C00295 | Pyrimidine metabolism |
| **2-hydroxyisovaleric acid** | C_5_H_10_O_3_ | 118.1311 | 1.58 | 3.29E-04 | ↓ | HMDB00407 | — | Synthesis and degradation of ketone bodies  Valine, leucine and isoleucine degradation |
| **taurine** | C_2_H_7_NO_3_S | 125.1469 | 1.48 | 3.67E-02 | ↑ | HMDB00251 | C00245 | Taurine and hypotaurine metabolism;  Tyrosine metabolism |
| **phosphate** | H_3_PO_4_ | 97.9952 | 1.12 | 4.66E-02 | ↑ | HMDB01429 | C00009 | — |
| **erythritol** | C_4_H_10_O_4_ | 122.1198 | 1.24 | 1.50E-02 | ↓ | HMDB02994 | C00503 | — |
| **oxidized dithiothreitol** | C_4_H_8_O_2_S_2_ | 152.235 | 1.25 | 1.66E-02 | ↑ | HMDB59664 | C01119 | — |
| **ribonic acid** | C_5_H_10_O_6_ | 166.1293 | 1.30 | 1.10E-02 | ↓ | HMDB00867 | C01685 | — |
| **2-hydroxyadipic acid** | C_6_H_10_O_5_ | 162.1406 | 1.51 | 1.78E-03 | ↓ | HMDB00321 | C02360 | — |
| **3-hydroxy-3-methylglutaric acid** | C_6_H_10_O_5_ | 162.1406 | 1.34 | 6.34E-03 | ↓ | HMDB00355 | C03761 | — |
| **1,5-anhydro-D-sorbitol** | C_6_H_12_O_5_ | 164.1565 | 1.44 | 2.00E-03 | ↓ | HMDB02712 | C07326 | — |
| **galacturonic acid** | C_6_H_10_O_7_ | 194.1394 | 1.38 | 2.58E-02 | ↓ | HMDB02545 | C08348 | — |
| **threitol** | C_4_H_10_O_4_ | 122.1198 | 1.17 | 4.38E-02 | ↓ | HMDB04136 | C16884 | — |
| **2-deoxy-D-ribotol** | C_8_H_16_O | 128.212 | 1.54 | 6.25E-04 | ↓ | HMDB31295 | C17145 | — |
| **oleamide** | C_18_H_35_NO | 281.4766 | 1.47 | 3.56E-03 | ↑ | HMDB02117 | C19670 | — |
| **iminodiacetic acid** | C_4_H_7_NO_4_ | 133.1027 | 1.08 | 4.68E-02 | ↑ | HMDB11753 | C19911 | — |

Note: ^a^Comparison of differential metabolites between chow diet group and HFD group with a Student’s *t* test; FC: fold change. VIP was obtained from OPLS-DA with a threshold of 1.0. The up and down arrows indicate a respective increased or decreased concentration of each metabolite in the chow diet group versus HFD group (chow diet / HFD).

**Table S3.** Endogenous metabolites of the liver from chow diet group and HFD group

| **Metabolites** | **Formula** | **Mol Weight** | **VIP** | **p-value** | **FC** | **HMDB** | **KEGG** | **Pathway(KEGG)** |
| --- | --- | --- | --- | --- | --- | --- | --- | --- |
| **pyruvic acid** | C_3_H_4_O_3_ | 88.0621 | 1.26 | 4.01E-02 | ↓ | HMDB00243 | C00022 | Glycolysis/Gluconeogenesis;  Citrate cycle(TCA cycle);  Pentose phosphate pathway |
| **gluconic acid** | C_6_H_12_O_7_ | 196.1553 | 1.73 | 8.53E-04 | ↓ | HMDB00625 | C00257 | Pentose phosphate pathway |
| **ribose-5-phosphate** | C_5_H_11_O_8_P | 230.1098 | 1.36 | 2.28E-02 | ↓ | HMDB01548 | C00117 | Pentose phosphate pathway; |
| **gama-aminobutyric acid** | C_4_H_9_NO_2_ | 103.1198 | 1.35 | 2.23E-02 | ↓ | HMDB00112 | C00334 | Alanine, aspartate and glutamate metabolism;  Arginine and proline metabolism;  beta-Alanine metabolism |
| **glutamic acid** | C_5_H_9_NO_4_ | 147.1293 | 1.53 | 6.75E-03 | ↓ | HMDB00148 | C00025 | Alanine, aspartate and glutamate metabolism;  Arginine and proline metabolism;  Taurine and hypotaurine metabolism |
| **ornithine** | C_5_H_12_N_2_O_2_ | 132.161 | 1.67 | 1.74E-03 | ↓ | HMDB00214 | C00077 | Arginine and proline metabolism |
| **galactonic acid** | C_6_H_12_O_7_ | 196.1553 | 1.34 | 2.43E-02 | ↑ | HMDB00565 | C00880 | Galactose metabolism |
| **glycerol** | C_3_H_8_O_3_ | 92.0938 | 1.40 | 1.71E-02 | ↓ | HMDB00131 | C00116 | Galactose metabolism;  Glycerolipid metabolism |
| **pyroglutamic acid** | C_5_H_7_NO_3_ | 129.114 | 1.74 | 7.26E-04 | ↓ | HMDB00267 | C01879 | Glutathione metabolism |
| **sarcosine** | C_3_H_7_NO_2_ | 89.0932 | 1.49 | 9.00E-03 | ↓ | HMDB00271 | C00213 | Glycine, serine and threonine metabolism |
| **cysteine** | C_3_H_7_NO_2_S | 121.1582 | 1.37 | 2.34E-02 | ↑ | HMDB00574 | C00097 | Glycine, serine and threonine metabolism;  Cysteine and methionine metabolism;  Taurine and hypotaurine metabolism |
| **pipecolinic acid** | C_6_H_11_NO_2_ | 129.157 | 1.59 | 4.19E-03 | ↓ | HMDB00070 | C00408 | Lysine degradation |
| **lysine** | C_6_H_14_N_2_O_2_ | 146.1876 | 1.83 | 1.89E-04 | ↓ | HMDB00182 | C00047 | Lysine degradation;  Lysine biosynthesis;  Biotin metabolism |
| **nonanoic acid** | C_9_H_18_O_2_ | 158.238 | 1.32 | 2.76E-02 | ↓ | HMDB00847 | C01601 | fatty acid |
| **2-hydroxybutyric acid** | C_4_H_8_O_3_ | 104.1045 | 1.31 | 2.99E-02 | ↓ | HMDB00008 | C05984 | fatty acid, monohydroxy |
| **cis-5,8,11-eicosatrienoic acid** | C_20_H_32_O_2_ | 304.4669 | 1.55 | 6.63E-03 | ↓ | HMDB10378 | C00219 | Long chain fatty acid |
| **eicosapentaenoic acid** | C_20_H_30_O_2_ | 302.451 | 1.72 | 1.09E-03 | ↓ | HMDB01999 | C06428 | Long chain fatty acid |
| **margaric acid** | C_18_H_30_O_2_ | 278.4296 | 1.70 | 1.31E-03 | ↑ | HMDB02259 | C06427 | Long chain fatty acid |
| **myristic acid** | C_14_H_28_O_2_ | 228.3709 | 1.36 | 2.19E-02 | ↑ | HMDB00806 | C06424 | Long chain fatty acid |
| **oleic acid** | C_18_H_34_O_2_ | 282.4614 | 1.79 | 3.25E-04 | ↑ | HMDB00207 | C00712 | Long chain fatty acid |
| **1-linoleoylglycerol** |  |  | 1.59 | 3.93E-03 | ↑ | — | — | lipid |
| **inosine** | C_10_H_12_N_4_O_5_ | 268.2261 | 1.28 | 3.53E-02 | ↓ | HMDB00195 | C00294 | Purine metabolism |
| **uridine-5'-phosphate** | C_9_H_13_N_2_O_9_P | 324.1813 | 1.43 | 1.53E-02 | ↓ | HMDB00288 | C00105 | Pyrimidine metabolism |
| **sucrose** | C_12_H_22_O_11_ | 342.2965 | 1.53 | 6.98E-03 | ↓ | HMDB00258 | C00089 | Starch and sucrose metabolism;  Galactose metabolism |
| **beta-sitosterol** | C_29_H_50_O | 414.7067 | 1.88 | 5.97E-05 | ↑ | HMDB00852 | C01753 | Steroid biosynthesis |
| **cholesterol** | C_27_H_46_O | 386.6535 | 1.79 | 4.24E-04 | ↑ | HMDB00067 | C00187 | Steroid biosynthesis;  Steroid degradation |
| **taurine** | C_2_H_7_NO_3_S | 125.1469 | 1.93 | 1.78E-05 | ↓ | HMDB00251 | C00245 | Taurine and hypotaurine metabolism |
| **tyrosine** | C_9_H_11_NO_3_ | 181.1885 | 1.66 | 2.23E-03 | ↓ | HMDB00158 | C00082 | Tyrosine metabolism;  Phenylalanine metabolism |
| **alpha-tocopherol** | C_29_H_50_O_2_ | 430.7061 | 1.45 | 1.28E-02 | ↓ | HMDB01893 | C02477 | Vitamin digestion and absorption; antioxidants |
| **campesterol** | C_28_H_48_O | 400.6801 | 1.94 | 9.70E-06 | ↓ | HMDB02869 | C01789 | A dietary phytosterol found in plants, lowering cholesterol |
| **lyxonic acid** |  |  | 1.41 | 1.61E-02 | ↓ | — | — | a sugar acid |
| **1-octadecanol** | C_18_H_38_O | 270.4937 | 1.94 | 1.17E-05 | ↓ | HMDB02350 | — | — |
| **3-hydroxyadipic acid** | C_6_H_10_O_5_ | 162.1406 | 1.63 | 2.80E-03 | ↑ | HMDB00345 | — | the oxidation of 3-hydroxy fatty acid |
| **4-deoxyerythronic acid** | C_4_H_8_O_4_ | 120.1039 | 1.30 | 3.22E-02 | ↓ | HMDB00498 | — | — |
| **dihydrocholesterol** | C_27_H_48_O | 388.6694 | 1.62 | 3.20E-03 | ↓ | HMDB00908 | — | — |
| **palmitelaidic acid** | C_16_H_30_O_2_ | 254.4082 | 1.73 | 7.85E-04 | ↑ | HMDB12328 | — | Long chain fatty acid |

Note: ^a^Comparison of differential metabolites between chow diet group and HFD group with a Student’s *t* test; FC: fold change. VIP was obtained from OPLS-DA with a threshold of 1.0. The up and down arrows indicate a respective increased or decreased concentration of each metabolite in the chow diet group versus HFD group (chow diet / HFD).

**Table S4.** Endogenous metabolites of the feces from normal chow diet group and HFD group

| **Metabolites** | **Formula** | **Mol Weight** | **VIP** | **p-value** | **FC** | **HMDB** | **KEGG** | **Pathway(KEGG)** |
| --- | --- | --- | --- | --- | --- | --- | --- | --- |
| **glucose-6-phosphate** | C_6_H_13_O_9_P | 260.1358 | 1.26 | 3.23E-02 | ↑ | HMDB01401 | C00092 | Glycolysis / Gluconeogenesis; |
| **pyruvic acid** | C_3_H_4_O_3_ | 88.0621 | 1.39 | 1.41E-02 | ↑ | HMDB00243 | C00022 | Glycolysis/Gluconeogenesis;  Citrate cycle(TCA cycle);  Pentose phosphate pathway |
| **malic acid** | C_4_H_6_O_5_ | 134.0874 | 1.21 | 4.02E-02 | ↑ | HMDB00156 | C00149 | Citrate cycle (TCA cycle) |
| **dodecanedioic acid** | C_12_H_22_O_4_ | 230.3007 | 1.42 | 1.15E-02 | ↑ | HMDB00623 | C02678 | fatty acid |
| **suberic acid** | C_8_H_14_O_4_ | 174.1944 | 1.36 | 1.79E-02 | ↑ | HMDB00893 | C08278 | fatty acid |
| **heptanoic acid** | C_7_H_14_O_2_ | 130.1849 | 1.20 | 4.29E-02 | ↑ | HMDB00666 | C17714 | fatty acid |
| **glutaric acid** | C_5_H_8_O_4_ | 132.1146 | 1.40 | 1.31E-02 | ↑ | HMDB00661 | C00489 | Fatty acid degradation;  Lysine degradation |
| **norleucine** | C_6_H_13_NO_2_ | 131.1729 | 1.40 | 1.70E-02 | ↑ | HMDB01645 | C01933 | fatty acid,monoamino |
| **5-hydroxyhexanoic acid** | C_6_H_12_O_3_ | 132.1577 | 1.29 | 2.68E-02 | ↑ | HMDB00525 | — | fatty acid,monohydroxy |
| **1-dodecanol** | C_12_H_26_O | 186.3342 | 1.50 | 7.21E-03 | ↓ | HMDB11626 | C02277 | Fatty alcohol |
| **1-eicosanol** | C_20_H_42_O | 298.5469 | 1.35 | 1.89E-02 | ↑ | HMDB11619 | — | fatty alcohol |
| **2-stearoylglycerol** | C_21_H_42_O_4_ | 358.5558 | 1.79 | 1.68E-04 | ↓ | HMDB31075 | — | lipid |
| **arachidonic acid** | C_20_H_32_O_2_ | 304.4669 | 1.45 | 8.59E-03 | ↓ | HMDB01043 | C00219 | Long chain fatty acid |
| **pentadecanoic acid** | C_15_H_30_O_2_ | 242.3975 | 1.22 | 4.17E-02 | ↑ | HMDB00826 | C16537 | Long chain fatty acid |
| **1-tetradecanol** | C_14_H_30_O | 214.3874 | 1.41 | 1.50E-02 | ↓ | HMDB11638 | — | Long chain fatty acid |
| **inosine** | C_10_H_12_N_4_O_5_ | 268.2261 | 1.57 | 3.00E-03 | ↓ | HMDB00195 | C00294 | Purine metabolism |
| **2'-deoxyguanosine** | C_10_H_13_N_5_O4 | 267.2413 | 1.23 | 3.69E-02 | ↓ | HMDB00085 | C00330 | Purine metabolism |
| **guanosine** | C_10_H_13_N_5_O_5_ | 283.2407 | 1.45 | 8.96E-03 | ↓ | HMDB00133 | C00387 | Purine metabolism |
| **uridine** | C_9_H_12_N_2_O_6_ | 244.2014 | 1.73 | 3.96E-04 | ↓ | HMDB00296 | C00299 | Pyrimidine metabolism |
| **lactic acid** | C_3_H_6_O_3_ | 90.0779 | 1.31 | 2.48E-02 | ↑ | HMDB00190 | C00186 | Pyruvate metabolism |
| **lanosterol** | C_30_H_50_O | 426.7174 | 1.37 | 1.71E-02 | ↑ | HMDB01251 | C01724 | Steroid biosynthesis |
| **cholesterol** | C_27_H_46_O | 386.6535 | 1.57 | 3.41E-03 | ↓ | HMDB00067 | C00187 | Steroid biosynthesis;  Steroid degradation |
| **indole-3-lactic acid** | C_11_H_11_NO_3_ | 205.2099 | 1.65 | 1.26E-03 | ↓ | HMDB00671 | C02043 | Tryptophan metabolism |
| **xanthurenic acid** | C_10_H_7_NO_4_ | 205.1669 | 1.46 | 9.12E-03 | ↓ | HMDB00881 | C02470 | Tryptophan metabolism |
| **5-hydroxyindoleacetic acid** | C_10_H_9_NO_3_ | 191.1834 | 1.87 | 1.59E-05 | ↓ | HMDB00763 | C05635 | Tryptophan metabolism |
| **tryptophan** | C_11_H_12_N_2_O_2_ | 204.2252 | 1.29 | 2.63E-02 | ↓ | HMDB00929 | C00078 | Tryptophan metabolism;  Glycine, serine and threonine metabolism |
| **3-hydroxyphenylacetic acid** | C_8_H_8_O_3_ | 152.1473 | 1.86 | 3.97E-05 | ↑ | HMDB00440 | C05593 | Tyrosine metabolism;  Phenylalanine metabolism |
| **2-ketoisocaproic acid** | C_6_H_10_O_3_ | 130.1418 | 1.31 | 2.22E-02 | ↑ | HMDB00695 | C00233 | Valine, leucine and isoleucine degradation |
| **3-methyl-2-ketovaleric acid** |  |  | 1.24 | 3.30E-02 | ↑ | HMDB00491 | C03465 | Valine, leucine and isoleucine degradation |
| **2-ketoisovaleric acid** | C_5_H_8_O_3_ | 116.1152 | 1.22 | 3.78E-02 | ↑ | HMDB00019 | C00141 | Valine,leucine and isoleucine degradation |
| **alpha-tocopherol** | C_29_H_50_O_2_ | 430.7061 | 1.40 | 1.27E-02 | ↑ | HMDB01893 | C02477 | Vitamin digestion and absorption |
| **chenodeoxycholic acid** | C_24_H_40_O_4_ | 392.572 | 1.67 | 9.72E-04 | ↓ | HMDB00518 | C02528 | Bile acid biosynthesis |
| **adipic acid** | C_6_H_10_O_4_ | 146.1412 | 1.22 | 4.29E-02 | ↑ | HMDB00448 | C06104 | Caprolactam degradation |
| **2-hydroxyglutaric acid** | C_5_H_8_O_5_ | 148.114 | 1.24 | 3.72E-02 | ↑ | HMDB00694 | C03196 | — |
| **methylsuccinic acid** | C_5_H_8_O_4_ | 132.1146 | 1.66 | 1.18E-03 | ↑ | HMDB01844 | C08645 | — |
| **2-deoxy-D-ribotol** | C_5_H_12_O_4_ | 136.1464 | 1.74 | 3.43E-04 | ↓ | HMDB33919 | — | — |
| **2-hydroxypyridine** |  |  | 1.52 | 5.20E-03 | ↑ | — | — | — |
| **2-ketobutyric acid** | C_4_H_6_O_3_ | 102.0886 | 1.47 | 7.78E-03 | ↑ | HMDB00005 | — | — |
| **4-Hydroxycyclohexylacetic acid** | C_8_H_14_O_3_ | 158.195 | 1.28 | 3.01E-02 | ↑ | HMDB00451 | — | — |
| **allocholic acid** | C_24_H_40_O_5_ | 408.5714 | 1.50 | 6.10E-03 | ↓ | HMDB00505 | — | — |
| **dihydrocholesterol** | C_27_H_48_O | 388.6694 | 1.21 | 4.42E-02 | ↑ | HMDB00908 | — | — |
| **lyxosylamine** |  |  | 1.25 | 3.44E-02 | ↑ | — | — | — |
| **pseudo uridine** |  |  | 1.63 | 1.74E-03 | ↑ | — | — | — |

Note: ^a^Comparison of differential metabolites between chow diet group and HFD group with a Student’s *t* test; FC: fold change. VIP was obtained from PLS-DA with a threshold of 1.0. The up and down arrows indicate a respective increased or decreased concentration of each metabolite in the chow diet group versus HFD group (chow diet / HFD).

**TABLE S5.** The weighted and unweighted unifrac significance test

| **Groups** | **WScore** | **Wsig** | **UWScore** | **UWsig** |
| --- | --- | --- | --- | --- |
| **Chow diet-HFD** | 1 | <0.0010 | 1 | 0.211 |
| **Untreated-Treated** | 1 | <0.0010 | 1 | 0.053 |
